# Identification, distribution and molecular evolution of the pacifastin gene family in Metazoa

**DOI:** 10.1186/1471-2148-9-97

**Published:** 2009-05-12

**Authors:** Bert Breugelmans, Gert Simonet, Vincent van Hoef, Sofie Van Soest, Jozef Vanden Broeck

**Affiliations:** 1 Department of Animal Physiology and Neurobiology, Zoological Institute K.U.Leuven, Naamsestraat 59, B-3000 Leuven, Belgium

## Abstract

**Background:**

Members of the pacifastin family are serine peptidase inhibitors, most of which are produced as multi domain precursor proteins. Structural and biochemical characteristics of insect pacifastin-like peptides have been studied intensively, but only one inhibitor has been functionally characterised. Recent sequencing projects of metazoan genomes have created an unprecedented opportunity to explore the distribution, evolution and functional diversification of pacifastin genes in the animal kingdom.

**Results:**

A large scale *in silico *data mining search led to the identification of 83 pacifastin members with 284 inhibitor domains, distributed over 55 species from three metazoan phyla. In contrast to previous assumptions, members of this family were also found in other phyla than Arthropoda, including the sister phylum Onychophora and the 'primitive', non-bilaterian Placozoa. In Arthropoda, pacifastin members were found to be distributed among insect families of nearly all insect orders and for the first time also among crustacean species other than crayfish and the Chinese mitten crab. Contrary to precursors from Crustacea, the majority of insect pacifastin members contain dibasic cleavage sites, indicative for posttranslational processing into numerous inhibitor peptides. Whereas some insect species have lost the pacifastin gene, others were found to have several (often clustered) paralogous genes. Amino acids corresponding to the reactive site or involved in the folding of the inhibitor domain were analysed as a basis for the biochemical properties.

**Conclusion:**

The absence of the pacifastin gene in some insect genomes and the extensive gene expansion in other insects are indicative for the rapid (adaptive) evolution of this gene family. In addition, differential processing mechanisms and a high variability in the reactive site residues and the inner core interactions contribute to a broad functional diversification of inhibitor peptides, indicating wide ranging roles in different physiological processes. Based on the observation of a pacifastin gene in Placozoa, it can be hypothesized that the ancestral pacifastin gene has occurred before the divergence of bilaterian animals. However, considering differences in gene structure between the placozoan and other pacifastin genes and the existence of a 'pacifastin gene gap' between Placozoa and Onychophora/Arthropoda, it cannot be excluded that the pacifastin signature originated twice by convergent evolution.

## Background

According to the NCBI platform (National Center for Biotechnology Information) more than 150 metazoan genome projects are in progress or completed and up to 36 million EST sequences are available . Comparative analysis of this huge dataset, covering numerous species of both vertebrates and invertebrates, has already proven to be a powerful tool to predict new gene products, facilitating their molecular characterization, illustrating cross species sequence homology, unraveling gene structures and revealing expansion or reduction of gene families during evolution. This report focuses on inhibitors of serine peptidases and more specifically on an inhibitor family of the S1 peptidases. According to the MEROPS peptidase database [1], the S1 family is, by far, the largest family of serine peptidases, comprising 144 known members in humans, 314 and 318 members in the two mosquito species *Aedes aegypti *and *Anopheles gambiae*, and 264 in *Drosophila melanogaster*. The number of identified peptidase inhibitors, however, is lower in insects than in humans. While in the MEROPS database 150 peptidase inhibitors are listed from humans, only 32, 11 and 42 have been annotated in the genomes of the Diptera *A. aegypti*, *A. gambiae *and *D. melanogaster*, respectively. In spite of this apparent discrepancy, previous studies have reported several members of the pacifastin peptide inhibitor family (I19 in the peptidase/inhibitor MEROPS database), all of which have been identified in arthropod species.

All pacifastin members that have been identified at the molecular level are precursor peptides sharing a common domain organization; an N-terminal signal sequence followed by a variable number of inhibitor domains. These domains are designated as PLDs (Pacifastin Light chain Domain) in reference to the light chain of pacifastin, the first member of the pacifastin family that was found in the crustacean species, *Pacifastacus leniusculus *[2,3]. All other pacifastin-related precursors (PP) that have been identified by cDNA cloning originate from only one other crustacean species, the Chinese mitten crab, *Eriocheir sinensis*, and from a few insect species belonging to the orders of Orthoptera and Hymenoptera [4-8]. However, previous *in silico *data mining studies have predicted additional pacifastin members in various other insect orders [9-11].

In crayfish, the pacifastin precursor consists of nine inhibitor domains or PLDs which are not processed into single domain peptides [3]. In insects, however, nearly all identified PPs are believed to be processed into smaller inhibitor peptides conforming to the presence of putative dibasic cleavage sites between inhibitor domains [5-8,12,13] and the detection of several insect (locust) PLD-related single domain peptides in the haemolymph [12].

The structural and biochemical characteristics of locust pacifastin-related inhibitors have been intensively studied by ^1^H NMR, crystallography, site-directed mutagenesis and activity studies [8,12-20]. All PLD-related domains have a characteristic conserved pattern of six cysteine residues (**Cys1 **– Xaa_9–12 _– **Cys2 **– Asn – Xaa – **Cys3 **– Xaa – **Cys4 **– Xaa_2–3 _– Gly – Xaa_3–6 _– **Cys5 **– Thr – Xaa_3 _– **Cys6**). Detailed analysis of the 3-D structure has shown that these six residues form three disulfide bridges (Cys1-4, Cys2-6, Cys3-5), giving members of the pacifastin family a typical fold and remarkable stability [16,18-21]. Each pacifastin-like inhibitor domain contains a reactive site (P1-P1') that is involved in the binding of the target peptidase and is located near the C-terminal end between the last two cysteine residues [20]. Inhibitor activity studies on the crayfish pacifastin and on locust pacifastin-like inhibitors have confirmed that the peptidase specificity is mainly determined by the residue at the P1-position [8,12-14,16,17,20]. Although pacifastin-related peptides share a common conformation, the family can be divided into at least two separate groups (I & II) based on different intramolecular interactions in the inner core region [22]. The variability of the reactive site residues (P1-P1') and of the inner core structure, as well as the occurrence of additional interaction sites, contribute to peptidase specificity and species selectivity of the pacifastin family members belonging to these two groups. Contrary to the detailed knowledge on structural and protease-inhibiting properties of locust pacifastin-like peptides, very little is known about their *in vivo *function(s) [9].

In crayfish, pacifastin itself is involved in the regulation of an immune response process (the prophenoloxidase cascade) [3,23-26]. In insects, however, there is no direct proof for a particular physiological function of the pacifastin family. Contrary to the situation in crayfish, locust pacifastin-like PI (SGPI- and SGPI-2) were not able to inhibit the endogenous prophenoloxidase activation cascade [27]. Nevertheless, detailed PP transcript profiling studies led to the suggestion that insect pacifastin-related peptides [6-8,27-29] may possess multiple functions as regulators of a wide diversity of serine peptidase-dependent processes [12,17,30].

This paper reports on a detailed, comparative *in silico *analysis of the sequences, the phylogenetic distribution and the domain organization of pacifastin-related precursors among Arthropoda and other phyla. In addition, based on a wide range of metazoan genome data, the structure, distribution and evolution of pacifastin genes among metazoan species were studied in an attempt to make predictions concerning the possible 'origin' of the ancestral pacifastin gene.

## Methods

### Database searches

In search of (novel) members of the pacifastin family in the available genome and EST databases, BLASTN and TBLASTN algorithms were performed, using a list of 16 known pacifastin-related precursor sequences from the Uniprot protein database as a query [9]. For both BLAST programs the expected threshold was adjusted to 0.1. In addition, for the TBLASTN program the BLOSUM62 matrix was used and a word size of 3 was selected when blasting PP sequences, while for the shorter PLD-like domains the word size was 2. For precursor sequences Detailed searches were performed on the vast number of nucleotide sequences available via the NCBI platform (National Center for Biotechnology Information) . As a complementary approach, various databases of individual species containing new sequences that are not yet incorporated into the NCBI platform were analyzed [see Additional file 1]. In addition, annotated databases as *Butterflybase *and *Ensembl *were searched via keywords and/or protein family accession numbers in clustered EST data and/or predicted gene structures. *In silico *predicted genomic sequences were manually verified using EST sequences (if possible). The identity of the newly predicted pacifastin-related members was further confirmed by scanning the *Interpro *protein database . This also provided a more detailed view on the domain organization of PP. The open reading frame (ORF) of the sequences and the presence of a signal peptide were predicted using BESTORF  and SignalP , respectively. To check and further complement previous search results, novel sequences were used as additional queries.

### Strategy

The data mining search was performed in a systematic way; covering as many phyla, classes and orders as possible. Per phylum of the animal kingdom (as defined by NCBI taxonomy), sequence data sets were analyzed for the presence of PP-encoding sequences and, whenever this was possible, additional searches were performed in subphyla, classes, orders and families of the phylum. All predicted PP-encoding sequences were assigned to simplified phylogenetic trees (see below) to obtain an overview of the presence of pacifastin-like peptides in numerous (sub)phyla, classes, orders, families and species, as well as to visualize the origin and evolution of the pacifastin gene family.

### Cloning of TCPP-1

Whole bodies of adult red flour beetles were added to reaction tubes containing 'Green Beads' (Roche, Indianapolis, IN, USA). Then, the samples were homogenized, total RNA was extracted and the resulting RNA was reverse transcribed as described by Breugelmans *et al*. (2008)[28]. Three different gene specific primers (U1: 5'-ATGAAGACCCTCATCCTGTG-3'; D1: 5'-ACAGTACACAGTAATTAGAG-3' and D1b: 5'-GGCTGGCAAACGGTATTTCT-3', SIGMA) were designed based on the genome predicted TCPP-1 sequence [GenBank: XM_966374]. PCR reactions, cloning and sequence analysis were preformed as described by Simonet *et al*. (2005)[8]. Subsequently, the nucleotide sequences were compared with genomic sequence data to analyze the gene structure as well as the precursor domain organization of the cloned sequences, as described below.

### Gene organization of pacifastin-related precursors

In combination with genomic sequences, EST-sequences were used to confirm the predicted ORF and to identify possible splice variants of the PP transcripts. To study the intron-exon organization of pacifastin genes in more detail, EST and/or cDNA sequences were aligned with genomic sequences using Splign . If no complete ORF was found, the Softberry splice finder tool (FSPLICE) was used to search the missing intron-exon junctions in pacifastin-related genes .

### Sequence comparison and phylogenetic analysis

Multiple alignments were performed on subsets of the obtained PLD-related domains  in order to compare amino acid sequences of the reactive site, the core region and other regions that might be involved in enzyme-binding and/or inhibitor stability. To study the evolution of pacifastin genes, simplified phylogenetic trees were composed with different metazoan taxa based on the most recent molecular phylogenomic data [31-38] and on the Tree-of-Life web project . Additional information on the classification system (phylum, class, order, family and genus) and on the number of known genomes, nucleotide and protein sequences was obtained from the NCBI taxonomy browser .

## Results and Discussion

### Novel pacifastin-related precursors

#### Identification of pacifastin-related precursor peptides

All sixteen pacifastin-related precursor peptide sequences from the Uniprot protein database were used as queries for an initial search of genomic/EST databases. Then, in search for additional PP-encoding sequences the query list was extended with newly found pacifastin-related sequences from other phyla, orders and classes.

A list of all identified PPs (83), the number of PLD-like domains per PP and the nature (genomic, EST, cDNA and protein) and accession numbers of the encoding sequences are provided as Supplementary data [see Additional file 2]. In contrast to previous studies, novel PPs were not only identified in many arthropods, but, for the first time, also in species belonging to other phyla. A pacifastin-related precursor was predicted in a non-bilaterian species belonging to the phylum of Placozoa, *Trichoplax adhaerens*. This precursor, named TAPP-1 according to the nomenclature of Simonet *et al*., (2002) [5], codes for 42 PLD-related inhibitor domains and is by far the longest member of the pacifastin family that has been identified so far. Another non-arthropod PP was found in the velvet worm, *Epiripatus sp*. [see Additional file 2]. Four PLD-related inhibitor peptides were predicted based on two sequences from a small cDNA library [39] of this onychophoran species. However, additional sequence information will be needed to verify whether (or not) these domains belong to the same precursor.

In line with previous findings, members of the pacifastin family appear to be present in a wide variety of insects. In addition to the earlier identified PPs [2,3,5-8,12,13,40], more than 70 new pacifastin-like precursors have been identified, distributed over 26 families covering nearly all insect orders. Interestingly, a pacifastin-like precursor was also discovered in a springtail species belonging to another class of Hexapoda, the Ellipura. This PP codes for three PLD-like inhibitor peptides.

Contrary to the wide distribution of pacifastin members in Hexapoda, previous studies had only identified two crustacean PP (pacifastin in the freshwater crayfish, *P. leniusculus*, and ESPP-1 in the Chinese mitten crab, *E. sinensis*) [2-4]. A detailed *in silico *search of the subphylum of Crustacea resulted in the prediction of seven new PPs, that altogether code for 22 PLD-like inhibitor domains, in six different species of various crustacean orders [see Additional file 2]. Thus, the pacifastin family appears to be widely distributed within Crustacea and ongoing or future genome projects are expected to reveal the existence of even more members of this family.

#### Domain organization of pacifastin-related precursors

All pacifastin-related precursors that have been identified so far, share a common domain organization that resembles the one of neuronal peptide precursors; they contain a signal peptide leading to the extracellular secretion of the mature protein, followed by one or more PLD-related inhibitor domains. Depending on the presence and the position of dibasic cleavage sites the secreted precursor peptide will subsequently be processed into smaller, single and/or multi domain inhibitor peptides.

The amino acid sequences of all previously and newly identified members of the pacifastin family are provided as supplementary data [see Additional files 3 and 4]. In total, 83 PP and 284 PLD-related inhibitor domains were analyzed. Even though in most species one or two PP have been found, the presence of additional PP in these or other species can not be excluded as their genome is not yet (fully) assembled and/or due to very few EST sequence data. Nevertheless, there appears to be considerable variation in the number of genes with a pacifastin-like signature in the assembled genomes of insect species, ranging from only one or two pacifastin genes [*e.g. ampp-1 *in the honey bee (*Apis mellifera*), *aapp-1 *in the yellow fever mosquito (*A. aegypti*), *agpp-1 *in the African malaria mosquito (*A. gambiae*), *tcpp-1 *and *tcpp-2 *in the red flour beetle (*T. castaneum*)] to at least 8 PP-encoding genes in the jewel wasp, *Nasonia vitripennis *[see Additional file 2]. Other examples of species that express a multitude of PPs are the locusts, *Schistocerca gregaria *and *Locusta migratoria *(7 and 4 known PPs, respectively).

Although the vast majority of PPs contain between 1 and 5 PLD-like domains, the number of domains per PP varies from 1 to 42 [*cf*. the PP from *T. adhaerens *(Placozoa)]. The number of PLDs in crustacean PPs varies between one in *Calanus finmarchicus*, and nine PLDs in the crayfish, *P. leniusculus*, while the variability in the number of PLD-related domains in insect PPs is even bigger; ranging from one in SGPP-2 of the desert locust, till nine and thirteen PLDs in the PPs of the dipteran, *Culex quinquefasciatus *and the caterpillar, *Bombyx mori*, respectively [see Additional file 2].

Previous studies showed that all multi-domain pacifastin-related precursors in insects contained dibasic cleavage sites between the PLD-like inhibitor domains, while no cleavage sites were found in pacifastin itself. To verify whether this characteristic is systematically different in these subphyla, and what is the situation in other phyla, the primary structure of all identified PPs was compared [see Additional file 3]. While dibasic cleavage sites appeared to be absent in PPs of crustacean species, with the exception of a PP in the flat porcelain crab, *Petrolisthes cinctipes*, they were found to be very common in insect PPs. In insects, nearly 90% of all predicted multi-domain PPs possess dibasic cleavage sites. In 60% of these PPs, PLD-related domains are separated by dibasic cleavage sites in such a way that a full post-translational processing would result in single domain inhibitor peptides. Although the processing of PPs is still hypothetical for most species (since the analysis is mainly based on *in silico *predictions), several locust PPs have already been shown to be cleaved into single domain inhibitors [12]. The apparent higher complexity of post translational processing of insect PPs may coincide with an additional level of regulation by prohormone convertases. Spatio-temporal differences in the expression of processing enzymes may therefore result in a more selective production of inhibitor peptides. Comparison of the primary structures of non-arthropod PPs revealed the presence of dibasic cleavage sites between sequential PLD-like domains of the PP found in Onychophora. In the PP of the more primitive placozoan species, *T. adhaerens*, that has 42 PLD-like domains, only one putative dibasic cleavage site was found.

Based on this information, it seems likely that pacifastin genes encoding multiple PLDs that are separated by dibasic cleavage sites already occurred before the divergence between Arthropoda and Onychophora, implying that most crustaceans PPs may have 'lost' these cleavage sites more recently, after their divergence from Hexapoda. Obviously, additional PP sequences of onychophoran and other more 'primitive' ecdysozoan species are needed to confirm this hypothesis.

#### Enzyme specificity of pacifastin-related inhibitor peptides

Each pacifastin-like inhibitor domain is composed of an exposed 'canonical' peptidase binding loop (P3-P3') (for nomenclature see Schechter and Berger [41]) that is anchored to the compact core region by two disulfide bridges. The 'canonical' loop contains the reactive site (P1-P1') that is involved in the binding of the target peptidase and is located near the C-terminal end between the last two cysteine residues [13,17,42]. The peptidase specificity of pacifastin-like inhibitors is, as in other canonical inhibitors, mainly determined by the residue at the P1-position. Inhibitor activity studies of locust pacifastin-related PI have shown that these peptides are serine peptidase inhibitors of either trypsin- or chymotrypsin-like peptidases according to the nature of the residue at the P1 position [8,12-14,16,17,20].

Comparison of the amino acid sequences of all predicted pacifastin family members revealed 17 different residues at the P1 and 14 at the P1' position, together forming as many as 74 different pairs [see Additional file 4]. The high variability of both residues of the reactive bond suggests that pacifastin-like PI cover a wide range of serine peptidase specificities. Based on the nature of the P1 residue, nearly half of all predicted metazoan PI (45%) are trypsin-like inhibitors, while 30% are predicted inhibitors of chymotrypsin-like peptidases. A small fraction (5%) contains a small, neutral amino acid (Gly, Ala and Val) at the P1 position corresponding to the interaction with a third type of serine peptidases, the elastase-like enzymes. Although the effect of an acidic or amide P1-residue (Asp, Glu, Asn and Gln) on the inhibitor's specificity is not yet known, 13% of all predicted PI contain such a residue. The remaining 7% also have unknown peptidase specificity, though the peptidase inhibitors with an unconventional Thr or bulky Met residue at the P1-position might be specific towards chymotrypsin-like peptidases.

In the 'most ancient' predicted pacifastin precursor (TAPP-1), 30 of the 42 PLD-related domains can be predicted as trypsin-like protease inhibiting, 4 PLDs can be expected to inhibit elastase-like enzymes, while the specificity of the remaining 8 PLDs is unknown. Noteworthy is the apparent absence of chymotrypsin-like enzyme inhibiting peptides in this phylum. In contrast, all PLDs of the PP from *Epiperipatus sp. (Onychophora) *appear to have chymotrypsin-like specificity. In Crustacea and Hexapoda the peptidase specificity of the pacifastin-related inhibitor peptides is more variable. In crustacean species, most multi-headed pacifastin-like inhibitor peptides contain the three types of specificity, suggesting that they can inhibit a broad spectrum of peptidases. As in crustaceans, these three PI specificity types are present in most insect orders. However, PI with specificity towards elastase-like enzymes are rare. In addition, a number of PI with unknown specificity was found in Diptera, Lepidoptera, Hymenoptera and Hemiptera (Supplement 4). The processing of insect PP into single or double domain inhibitor peptides with a high variability in enzyme specificity is in line with the idea that insect pacifastin-related members play a role in a variety of physiological processes.

#### Gene structure

**Figure 1:**
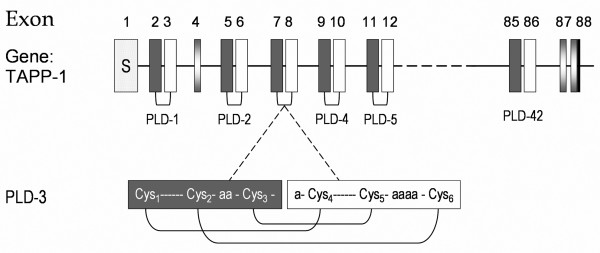
**Gene structure of the *tapp-1 *gene of *Trichoplax adhaerens***. The upper part of the figure visualizes the *tapp-1 *gene structure. The 88 exons are depicted as boxes and numbered accordingly, while the horizontal connecting lines represent the intron regions. The first box corresponds to the exon that codes for the signal peptide (S). The exons in grey and white encode the respective first and last half of a particular PLD-related domain. Exons 4, 87 and 88 correspond to regions of the *tapp-1 *gene that do not code for PLD-related inhibitor domains. The last box (exon 88) contains the stop codon (shown in black). The bottom part of the figure shows in more detail how the combination of two exons (7 and 8) is needed to form one PLD-related domain (e.g. PLD-3). For every 'exon-pair', the first exon codes for the first three cysteine residues of the PLD-related domain (in grey), while the last three cysteines are encoded by the second exon (in black). The diagram underneath illustrates the topography of the three disulfide bridges stabilizing the typical pacifastin-like fold.

Simonet *et al*. (2003) were the first to report the gene structure of a pacifastin-related member in the African malaria mosquito, *A. gambiae *[11]. The growing amount of assembled insect genomes has led to the prediction of more pacifastin-related genes in insects. However, these predictions are the result of automated genome sequence analyses and are not always accurate. Therefore, the PP gene structures of six insect species from five different orders (Coleoptera, Diptera, Hemiptera, Hymenopteran and Phthiraptera) and of the 'ancient' placozoan *Trichoplax *PP were analyzed and compared. In all except the latter species, EST sequences were used to verify the predicted gene structure. The *tapp-1 *gene of *T. adhaerens *has a remarkable intron/exon architecture (Figure 1). The gene consists of 88 exons, and is the most complex pacifastin-like gene studied so far. The signal peptide is encoded by the first exon, while each of the 42 PLD-like inhibitor domains is coded for by two neighboring exons as illustrated in Figure 1. Each exon of the *Trichoplax pp *gene codes for nearly half of the PLD-like domain including three cysteine residues. As a result, only when both exons are combined three S-S-bonds, critical for the PI conformation, can occur.

Also in insect PP-encoding genes, the first exon codes for a signal peptide. One exception is the signal peptide of NVPP-1 that is encoded by two neighboring exons. Contrary to the *Trichoplax tapp-1 *gene, a single PLD-like domain or even multiple PLDs are coded for by one exon. This observation indicates that exon duplications, whether or not followed by in-frame exon fusion, are one of the evolutionary events that may have led to the formation of more complex pacifastin genes, encoding PPs with multiple inhibitor domains combining two exons into a single PLD-like domain encoding exon. Despite this general characteristic for insect PP genes, there are some variations in the intron/exon structure and transcript splicing.

**Figure 2:**
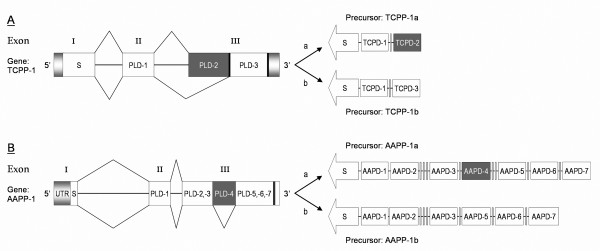
**Gene structure and alternative splicing of the *tcpp-1 *gene (A) and the *aapp-1 *gene (B)**. The left part of the figure visualizes the *tcpp-1 *and *aapp-1 *gene structure; the silver boxes correspond to the untranslated regions, the three exons are indicated as white and grey boxes, while the horizontal connecting lines represent the intron regions. In both genes, the third exon encodes multiple PLD-related domains (grey and white boxes) and contains a stop codon (pictured in black). The right part of the figure visualizes two splicing scenarios (a and b) that finally lead to two different pacifastin-related precursor peptides. **(A) **Splicing of the *tcpp-1 *gene according to ***scenario a ***results in a precursor polypeptide that codes for a signal peptide (white arrow), and two PLD-related domains visualized by a white and a grey box (TCPD-1 and TCPD-2, respectively). In ***scenario b***, differential splicing of the *tcpp-1 *gene results in a precursor polypeptide with the same signal peptide and PLD-1 domain, but a different second pacifastin-like inhibitor domain (TCPD-3). In both precursors, the two domains are separated by putative dibasic cleavage sites (grey small bars). Full processing of both PP variants results in single-domain inhibitor peptides. **(B) **According to ***scenario a***, all 7 PLD-related peptide encoding sequences of the *aapp-1 *gene are transcribed. In ***scenario b ***on the other hand, an additional splicing occurs in the third exon, resulting in the removal of the fourth PLD-related domain (grey box). In both precursors, all domains except PLD-1 and PLD-2 are separated by putative dibasic cleavage sites (grey small bars). Upon processing this results in the cleavage into one double-headed inhibitor peptide (PLD-1 and PLD-2) and 6 or 5 single inhibitor domain peptides.

Two pacifastin-like genes (*tcpp-1 *and *tcpp-2*) are present in the genome sequence of the red flour beetle, *T*. *castaneum*. The *tcpp-1 *gene is annotated to code for a single PP containing three PLD-related domains [GenBank: XM_966374]. Analysis of the gene structure shows that the first exon codes for a signal peptide and that the second and third exon code for one and two PLD-like domains, respectively. However, manual analysis of additional EST sequence data revealed two splice variants of the TCPP-1 transcript, both of which are different from the annotated transcript. To verify our observation, primers were designed to amplify both predicted splice variants. Using the primer set (U1-D1) two fragments of different length were amplified by means of RT-PCR. In addition, a second reverse primer (D1b) was derived to specifically amplify the presumed shorter variant. Analysis of the different PCR products, confirmed the occurrence of a short (279 bp) and a long (399 bp) transcript. Further sequence analysis showed that the difference between both variants is due to differential splicing of the third exon of the *tcpp-1 *gene. The (long) transcript codes for a pacifastin-related precursor (TCPP-1a) that contains a signal peptide and two PLDs (TCPD-1 and TCPD-2). Due to a stop codon at the end of the TCPD-2 encoding sequence, the third PLD is not translated (Figure 2A: splicing scenario a). The TCPP-1b-mRNA, on the other hand, is shorter because the first part of exon III (encoding TCPD-2) is spliced out (Figure 2A: splicing scenario b).

Splice variants of PP-encoding genes are also found in mosquito species (Diptera) and seem to be a more common phenomenon for the insect members of the pacifastin family. In *A. aegypti*; a 'manual' EST analysis of the *aapp-1 *gene revealed the existence of at least two transcript variants (Figure 2B). Because splice variants are confirmed with EST sequences, the lack of splice variants in the other studied insect *pacifastin *genes can be due to a limited availability of EST sequence data. The previously incorrect prediction of TCPP-1 shows that, in some cases, conventional cloning techniques and 'manual' corrections based on ESTs are necessary for a correct annotation. This is also illustrated by the automated prediction of the *agpp-1 *gene of *A. gambiae*: while the previously predicted gene product only contained three PLDs, our 'manual' analysis of the genomic sequence has revealed a more complete ORF encoding 4 PLD-like domains.

**Figure 3:**
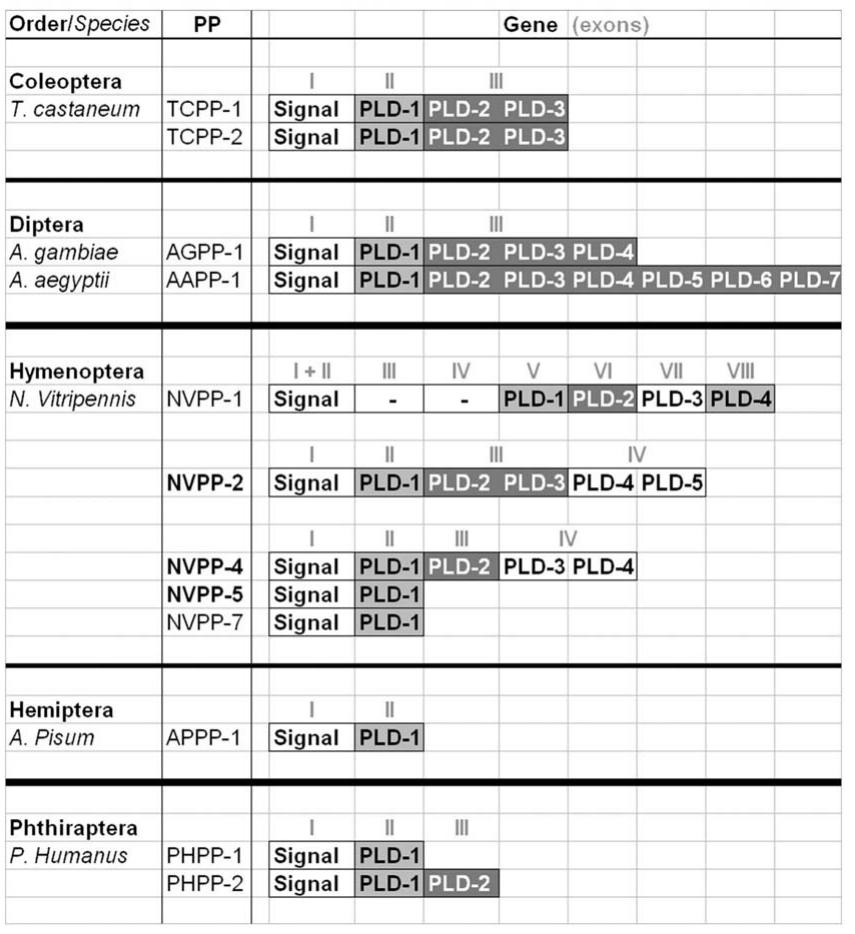
**Gene structure (exons) of coleopteran, dipteran, hymenopteran, hemipteran and phthirapteran pacifastin-like genes**. For each precursor (PP) gene, the exons are shown as roman numbers. The exons that encode the signal peptide and different PLD-related domains of the PP are illustrated by white and grey boxes. The PP **indicated in bold **are encoded by clustered genes.

While *A. aegypti*, *A. gambiae *and the hemipteran pea aphid, *Acyrthosiphon pisum*, contain only one pacifastin gene, other insect species (Coleoptera, Hymenoptera and Phthiraptera) have multiple pacifastin gene copies (Figure 3). These genes are often clustered, indicating recent gene duplication events. In case of the Jewel wasp, *N. vitripennis*, at least eight pacifastin genes are predicted in the genome (Supplement 1). The *nvpp-2, -4 *and -*5 *genes are clustered, as are the *nvpp-1, -3*, and -*8 *genes and the *nvpp-6 *and -*7 *genes [GenBank: NW_001815570, NW_001817237 and NW_001815682 respectively]. EST sequence data are available for the *nvpp-1, -2, -4, -5 *and -*7 *genes and more in depth analysis showed that the *nvpp-1 *gene has a rather exceptional gene structure (Figure 3). This gene contains eight exons and codes for four PLD-like domains. In contrast to all other insect PP genes, the signal peptide is encoded by two neighboring exons instead of one. In addition, the exons III and IV of the *nvpp-1 *gene code for an internal fragment that does not contain a PLD-like signature. For the *nvpp-3*, *nvpp-6 *and *nvpp-8 *genes no EST sequence data are available. FGENESH analysis of the *nvpp-8 *genomic sequence [GenBank: XM_001601664], however, suggests that the exon 3–4 junction was not correctly predicted. Instead of one gene composed of 5 exons encoding a PP with a signal peptide and four PLDs, it is more likely that the last exon is part of an additional PP gene. More EST sequence data and/or cDNA cloning are needed to clarify this hypothesis.

These data indicate that recent gene and/or exon duplication events in combination with the occurrence of splice variants underlie the considerable differences in the number of PP/PLD between different insect species, varying from a single PP with only one PLD in *A. pisum *to 8 PPs with more than 20 PLDs in *N. vitripennis*.

### Origin and evolution of pacifastin-like peptides

#### Pacifastin-like peptides in Metazoa

Based on previous *in silico *data mining studies it was hypothesized that pacifastin-related peptides were limited to arthropods and in particular to insects and crustacean species [5-8,12,13]. However, this study is the first to report the occurrence of pacifastin members in two other phyla, including the Placozoa. In the animal kingdom, the Bilateria (containing phyla such as Arthropoda, Chordata, Annelida and Mollusca) are the most 'evolved' clades. Whole-genome phylogenetic analyses suggest that Placozoa, that contain only one species, *T. adhaerens*, represent a sister group to all other Eumetazoa (Cnidaria and Bilateria) and form a separate clade [43].

The 'primitive' placozoan species, *T. adhaerens*, is a small, flat and disc-shaped (1–2 mm) animal having only four morphologically different somatic cell types, excluding nerves and sensory or muscle cells [44]. Surprisingly, genome analysis has shown that the relatively small genome of this 'primitive' animal encodes the basic machinery (e.g. with a rich repertoire of transcription factors and signaling pathways) required for complex developmental processes and neural/muscle differentiation that is typically associated with Bilateria [43]. Based on these observations, on mitochondrial sequencing analysis and on particular morphological characteristics of *T. adhaerens*, it is very likely that Placozoa appeared rather late in metazoan evolution and are more closely related to Bilateria than previously expected based on their primitive morphology [43,45-50].

**Figure 4:**
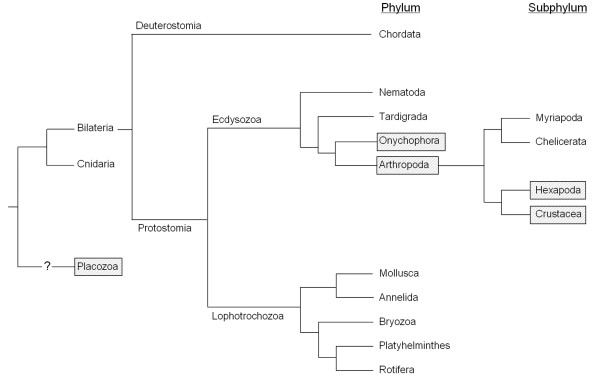
**Simplified phylogenetic tree of metazoan (sub)phyla**. Only the (sub)phyla that hold more than 1000 nucleotide or EST sequences according to the NCBI taxonomy platform were taken into account. The (sub) phyla, in which pacifastin-related members were predicted, are highlighted. A question mark is used when the origin of the (sub) phylum is uncertain.

No pacifastin-related sequences were found in Deuterostomia, nor in Annelida and Mollusca, the best characterized lophotrochozoan phyla (Figure 4). Unfortunately, very few sequence data are available of the remaining lophotrochozoan phyla and, therefore, it is not clear whether pacifastin-like genes are entirely absent in Lophotrochozoa. In addition, no significant hits were found in the ecdysozoan phyla, Tardigrada and Nematoda, despite the large amount of available sequence data from the latter phylum. However, the occurrence of pacifastin related genes in Tardigrada cannot be excluded, since sequence information is scarce in this phylum. Bilaterian pacifastin-like genes have been found in Panarthropoda, more specifically in the sister phyla, Arthropoda and Onychophora.

Based on the phylogenetic position of Placozoa, the PP endocing gene found in *Trichoplax *can be considered as the closest relative of the ancestral pacifastin gene, suggesting that the pacifastin genes of bilaterian species evolved by in-frame exon-fusion. However, since pacifastin genes have only been found in two related bilaterian phyla, this hypothesis would imply that during bilaterian divergence the pacifastin gene was 'lost' in the evolutionary lineages leading to Deuterostomia and to most phyla belonging to the Lophothrochozoa and Ecdysozoa. Therefore, a second hypothesis, which suggests that the pacifastin signature may have arisen twice by convergent evolution, appears equally plausible. According to this scenario, one pacifastin gene has emerged rather early in animal evolution and evolved to the currently known pacifastin gene in *Trichoplax*. Independently, the 'ancestor' of the *pacifastin *genes in Panarthropoda may have emerged within the ecdysozoan clade after the divergence from Nematoda and before the split between Arthropoda and Onychophora.

#### Pacifastin-like peptides in Arthropoda

The phylum of Arthropoda contains the highest number of animal species on Earth, with more than one million species described. To investigate the distribution and evolution of the pacifastin-related genes in this monophyletic group, we searched for new pacifastin members in the four subphyla: Chelicerata (horseshoe crabs, spiders and pycnogonids), Myriapoda (centipedes, millipedes, etc.), Hexapoda (insects and springtails) and Crustacea (shrimps, crabs, etc.). In the two sister groups, Chelicerata and Myriapoda, no pacifastin-like genes were found (Figure 4). However, the presence of pacifastin members in these subphyla can not be fully excluded, since the amount of available sequence data is still limited. On the other hand, precursor peptides with the 'pacifastin signature' were discovered in the two remaining subphyla, Crustacea and Hexapoda (Figure 4).

Based on the presence of pacifastin-like genes in both Arthropoda and Onychophora, the common ancestor of the Panarthropoda most likely possessed a pacifastin-like gene. Consequently, the Chelicerata and Myriapoda may have 'lost' this gene after their divergence from the other Arthropoda, estimated less than 600 million years ago. This observation is in line with both phylogenomic data (ribosomal sequences, mt genome, *hox *genes, EST sequences) and morphological characteristics (similarity in a neurogenesis developmental mechanism), suggesting that Myriapoda and Chelicerata constitute a separate clade (the Paradoxopoda) from the Hexapoda and Crustacea clade (the Pancrustacea) [31,51-54].

#### Pacifastin-related peptides in Crustacea

**Figure 5:**
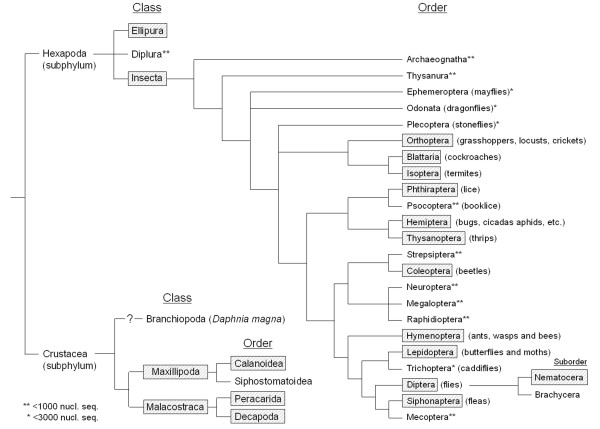
**Simplified phylogenetic tree of the different classes and orders of Hexapoda and Crustacea**. Orders, for which less than 3000 and 1000 nucleotide or EST sequences are deposited in Genbank, are marked by an * and **, respectively. The classes and orders, in which pacifastin-related members were identified, are highlighted.

More than twenty years ago, the crayfish, *Pacifastacus leniusculus*, was the first crustacean in which a pacifastin member was identified [2,3]. Contrary to the subsequent identification of many PPs in insects, only two crustacean PPs had been reported. Several genome projects of economically important shrimp, lobster and crab species currently generate a growing amount of sequence data, showing that pacifastin members are more widely spread in this phylum (Figure 5). In the class of Maxillopoda, a zooplankton species (*C. finmarchicus*) expresses a pacifastin-like precursor peptide [see Additional file 2]. Nevertheless, no pacifastin-like sequences have been found in the related order of Siphonostomatoida, despite the large amount of available EST sequences. In the large class of Malacostraca, pacifastin-like sequences were found in several species of Peracarida and Decapoda, two orders for which a significant amount of nucleotide sequences are available. Most of these species are commercially important, such as the Common freshwater shrimp (*Gammarus pulex*), the Pacific white shrimp (*Litopenaeus vanmei*) and the American lobster (*Homarus americanus*). Remarkably, pacifastin-like sequences appear to be absent in the class of Branchiopoda. The more than 200,000 ESTs and the fully sequenced genome of *Daphnia pulex *do not contain any pacifastin-like transcripts or genes, suggesting that the pacifastin gene was 'lost' in the *Daphnia *lineage, and, maybe, in the entire class. Noteworthy is the uncertain phylogenetic relationship of this class to other pancrustaceans. Recent phylogenetic studies suggested that some crustacean lineages, namely Malacostraca and Branchiopoda, are sister groups to Hexapoda, with the exclusion of other crustaceans, and that insects frequently emerge as a nested clade within Crustacea, which may constitute a paraphyletic group [55-59].

#### Pacifastin-related peptides in Hexapoda

The subphylum of Hexapoda is currently subdivided in three important classes, the Ellipura or springtails (Collembola), the Diplura or two-pronged bristletails and the Insecta (Figure 5). The presence of pacifastin genes in insects has been proven in several previous studies, but for the first time a pacifastin member was found in the springtails and more precisely in *Folsomia candida*. This springtail of the family Isotomidae is blind, unpigmented and parthenogenetic as some insect species, such as aphids, some bees and parasitic wasps. On the contrary, no sequences encoding the pacifastin-like signature were found in Diplura. This apparent absence may be due to the lack of available sequence data for this class (less than 1000 nucleotide sequences). On the other hand, pacifastin family members were found in the majority of insect orders; from heterometabolous (hemimetabolous) orthopteran, blattarian and isopteran species to holometabolous hymenopteran, lepidopteran, dipteran and siphonapteran species. In these orders, members of the pacifastin family occur in nearly all branches, while their absence in the remaining insect orders may be accounted for by the low amount of available nucleotide sequences (Figure 5). In Hemiptera, PPs are found in all three important clades, *i.e*. the clade of the true bugs (Heteroptera), the cicadas (Auchenorrhyncha) and the aphids (Sternorhyncha). Also in Lepidoptera, PPs were identified in both butterflies and moths and in all other superfamilies of the Neolepidoptera. There is one remarkable exception in the order of Diptera or 'true flies'. Contrary to the Suborder of the Nematocera that contains many species expressing PPs (mosquitoes, sand flies and other bloodsucking flies), no pacifastin-related members have been identified in the suborder of the Brachycera (Figure 5). This large suborder contains a huge variety of highly specialized dipteran species. However, the available sequence data are mainly restricted to the family of Drosophilidae and a few other species such as the horn fly (*Haemotobia irritans*) and the Mediterranean fruit fly (*Ceratitis capitata*). Whether the pacifastin gene is 'lost' in the entire suborder of the Brachycera, as it seems the case for the family of Drosophilidae, can therefore not yet be confirmed.

Another noteworthy observation is the variation in the number of pacifastin genes in Hymenoptera. Only one PP-encoding gene is present in the genome of the honey bee, *Apis melifera*, while at least eight PPs were found in an endoparasitoid wasp, *Nasonia vitripennis*. Based on the function of pacifastin as a regulator of the innate immune system in the crayfish [26], it seems rational to assume that some pacifastin-related inhibitors might play a similar role in insect immunity. The expression of multiple pacifastin PPs, and their processing into several pacifastin-like inhibitors, suggests that at least some pacifastin-related PI may have additional functions. In the case of *N. vitripennis*, pacifastin gene duplications and adaptive evolution might be correlated with a functional diversification of (some) pacifastin-like PIs as repressors of host immune systems. In analogy, a pacifastin-related inhibitor was identified in the venom of a closely related endoparositoid wasp (*Pimpla hypochondriaca*) and this venom includes different compounds that are known to interfere with the immune system of the host organism [60,61].

## Conclusion

This paper is the first to report on a large scale *in silico *study of the pacifastin family. The primary structures of all identified PPs and PLDs were analyzed, as well as the organization of pacifastin genes. In addition, sequences were manually corrected, splice variants were determined and gene structures were verified, whenever possible, using EST sequences and/or conventional cloning techniques. By this combined approach pacifastin members were identified in 55 species, divided over three distinct animal phyla. Within Arthropoda, PPs were identified in Insecta, Ellipura and Crustacea, suggesting a wide distribution of members of the pacifastin family in these branches of the phylum. An additional PP showing high sequence similarity with some arthropod PPs was identified in Onychophora. Surprisingly, an exceptionally large PP was predicted in the genome of the placozoan, *T. adhaerens*. Genome analysis and comparison of the amino acid sequences suggested that the *Trichoplax *gene may constitute an ancestral gene to the pacifastin family members present in arthropods and onychophorans. However, it can not be excluded that the pacifastin signature may have originated twice during metazoan evolution. Detailed analysis of the domain and gene organization of different insect PPs, indicated that gene duplication, exon duplication (PLD-like domain duplication) and alternative splicing of the corresponding transcripts have occurred during insect evolution. Together with a high variability of the residues of the reactive site, the different core types and the presence of putative cleavage sites between PLDs, these evolutionary events seem to be linked with adaptive functional diversification of this family, in response to differences in the ecological niches of the insects.

For many species, none or very few sequence data are available. However, ongoing genome and EST projects are expected to generate additional sequence data and to lead to an even more detailed view on the phylogenetic distribution and evolution of the pacifastin gene family in Metazoa.

## Authors' contributions

BB carried out the *in silico *and molecular analyses and produced a draft of the manuscript. GS conceived and coordinated the study and helped to draft the manuscript. VvH helped with bioinformatics analysis and gathering of data. SVS participated in sequence alignment and in the molecular biological analyses. JVB is the senior author who supervised the work and critically revised the manuscript. All authors have read and approved the final manuscript.

## Supplementary Material

Additional file 1 **Databases of invertebrate genomes**. This table summarizes the databases that were used for the data mining searches Click here for file

Additional file 2 **Overview of all identified and predicted pacifastin-related precursors according to their phylogenetic classification; (sub)phylum, family and species**. Species of which a complete or partially assembled non mitochondrial genome is known are indicated by an | and species, in which the pacifastin gene structure was studied, are marked in red. For each pacifastin-related precursor the number of the PLD-related domains is given. In addition, the molecular nature of the PP-(encoding) sequences and the associated accession numbers are given. Partial sequences (ORF or protein) are indicated by an *. Click here for file

Additional file 3 **List of all previously and newly identified pacifastin-related precursors in Metazoa**. The amino acid sequences of PP are given in FASTA format and categorized according to the species' classification (family, order, phylum and regnum). The conserved cysteine residues are highlighted in grey, while the putative signal peptide is underlined and possible dibasic cleavage sites are highlighted in black. Click here for file

Additional file 4 **List of all previously and newly identified PLD-related inhibitor domains in Metazoa**. The amino acid sequences of PLD-like domains are given in FASTA format and categorized according to the species' classification (family, order, phylum and regnum). The conserved cysteine residues are highlighted in grey, while the catalytic residues of the reactive site (P1-P1') are highlighted in black. Click here for file
