# Supplementary material for: Identification, distribution and molecular evolution of the pacifastin gene family in Metazoa
Source: BMC Evol Biol. 2009 May 12;9:97. doi: 10.1186/1471-2148-9-97 (PMC2689174; doi:10.1186/1471-2148-9-97)
Supplement: Additional file 1 — Databases of invertebrate genomes. This table summarizes the databases that were used for the data mining searches [file 1471-2148-9-97-S1.pdf]

(Sub)phylum	Order	Database
Hexapoda	Hymenoptera	• http://www.hgsc.bcm.tmc.edu/projects/honeybee<br>• http://racerx00.tamu.edu/blast/blast.html
	Diptera	• http://www.ensembl.org<br>• http://flybase.bio.indiana.org<br>• http://www.vectorbase.org<br>• http://www.genedb.org
	Lepidoptera	• http://210.249.76.231/cgi-bin/lib_ptoc_MX<br>• http://papilio.ab.a.u-tokyo.ac.jp/Bombyx_EST/<br>• http://heliconius.cap.ed.ac.uk/butterfly/db/<br>• http://morus.ab.a.u-tokyo.ac.jp/cgi-bin/index.cgi<br>• http://sgp.dna.affrc.go.jp/KAIKObase/<br>• http://silkworm.genomics.org.cn/
	Coleoptera	• http://beetlebase.org/
	Hemiptera	• http://urgi.versailles.inra.fr/projects/Aphid
Crustacea	Branchiopoda<br>Copepoda<br>Malacostraca	• http://iubio.bio.indiana.edu:7182/blast/
Nematoda		• http://www.wormbase.org/<br>• http://www.nematode.net/)
