# Supplementary material for: Identification, distribution and molecular evolution of the pacifastin gene family in Metazoa
Source: BMC Evol Biol. 2009 May 12;9:97. doi: 10.1186/1471-2148-9-97 (PMC2689174; doi:10.1186/1471-2148-9-97)
Supplement: Additional file 3 — List of all previously and newly identified pacifastin-related precursors in Metazoa. The amino acid sequences of PP are given in FASTA format and categorized according to the species' classification (family, order, phylum and regnum). The conserved cysteine residues are highlighted in grey, while the putative signal peptide is underlined and possible dibasic cleavage sites are highlighted in black. [file 1471-2148-9-97-S3.pdf]

Placozoa - Metazoa

Trichplax adhaerens

>TAPP-1
MEMIIPIPTMTKRSLQYKQLFTGTLHIFALELVIFSIIDFQATNKDFNAVLSTACKFNNINYQVGENRKAEDGCN
TCMCLKNGGWGCTRMACQPTGTNNLPRCPFNPQDNRICPMMVTTFCKRDNCSQCCENSNGCNPNEELLVRRDQCN
NGTKPTRPNGTDTCIFEEKLFRLGETVKQKCNQCKCLGQNKWACTRIACPDTPNHPDTTKLCVHNTMLLKQNETK
RKDCNTCKCLGSNRWACTRMFCPPPEGRRDVCIFEGKMFRKEETRADDCNICRCFGNNRWGCTRKACPPREFTCN
YGGRRFRTGDYVARGCNVCRCTPTGKFMCTRNDCNDRPDTPPKTRKCILNDREYNISAVTKIDCNYCRCLKGIWA
CTRMACQPTNQTNMLDNVCEKDGRFFVRGELRREKCNLCRCSNSGRWMCTTKMCPPEFNLSNATVPQCQHKTRTF
FHGEIRKDQCNFCNCTRNETWACSNYSCGYPYSQEGCVHRGRRFNIGHIKKQSCNTCKCLTNGGWGCTRMMCPPN
RPNTTCQMGNEVSRVGETKFLDCNKCTCSSTGKWMCTEKVCVEEKYKHISITFYLVIPSRSFSNLSLDVTRVCKF
NNQYFQKGEQIRNSCNLCKCGYTGQWACTRMMCNPNYEKVELIGFTNDSPMTMVLMLESLPQCTYQNTTLYLGEV
RKFNCSYCSCRGNETVECMELPCAREESPVCYHMGTTYPTGFIAKKDSCNKCKCNKYGLWACTAFKCPKQPMATK
LCTHNNEIYRPSEIRMDDCNRCMCLKDGAWGCTRMMCPPRNETTPTCTFRGRTFAAGTIKKQGCNMCKCYTNGEW
NCTTKPCSNPHVKFICEMRGMFLFHGETIRKNCNLCTCLPTGNMACTAKDCTENETLSETTCKHGKRLFYEGERS
KQDCNECQCLSNGSWNCTQNTCENKPERKCRMMDRTFEVGHRMRHRCRHCRCMEGGFWGCRHRPCRHHENTTARV
CRHSGLKFNHGENRSLDCNTCICNNGSWACTMKMCPPGSEICVHNNEYYSHRETRKQDCNTCMCTNGKWACTRMM
CPPKRKSCFHENETFQHGATRKQDCNTCKCIDGRWGCTRMMCPPKEEVCQYKDEMFQQGEITKQDCNTCKCIKGR
WACTRMLCPPREGVCEYEDTIYEHNETRKQDCNTCKCITGKWACTRMMCPPKEEREVCTFRDTLYEHGETRKQDC
NTCKCITGKWACTRMMCPPKEEVCQYKDTTFAHGETTKQDCNTCKCIKGRWACTRMLCPPREGVCEYEDTIYEHN
ETRKQDCNTCKCITGKWACTRMMCPPKEEREVCTFRDTLYEHGETRKQDCNTCKCITGKWACTRMMCPPKEEVCQ
YKDTTFAHGETTKQDCNTCKCIKGRWACTRMLCPPREGVCEYEDTIYEHNETRKQDCNTCKCITGKWACTRMMCP
PKEEREVCTYRDTLYEHGETRKQDCNTCKCITGKWACTRMMCPPKEEVCQYKDTTFAHGETTKQDCNTCKCIKGR
WACTRMLCPPREGVCEYEDTIYEHNETRKQDCNTCKCITGKWACTRMMCPPKEEREVCTYRDTLYEHGETRKQDC
NTCKCITGKWACTRMMCPPKEEVCQYKDTTFAHGETTKQDCNTCKCIKGRWACTRMLCPPREGVCEYEDTIYEHN
ETRKQDCNTCKCITGKWACTRMMCPPKEEVCQYKDTTFAHGETTKQDCNTCKCIKGRWACTRMLCPPREGVCEYE
DTIYEHNETRKQDCNTCKCITGKWACTRMMCPPKEEREVCTYRDTLYEHGETRKQDCNTCKCITGKWACTRMMCP
PNPLAPCVYEKKNYNITAVVEKDCQRCICGLFARWYCQRKPNCTPTIPRNVTCALGSQQVPTGYKMEEKCRQCTC
MNDGQWKCNVNSECLKPKFQNCTLTNGQIIPHGHVEEKKCEKCKCNDGKYVCTNKPCPLETNTCEYQNKKFPVNT
VKLIGLCHRCKCSPAGNWRCLDLGCNSNNNANNLPNEHIIVRPDSIF

Onychophora -Meatzoa

***Epiperipatus sp.* – Peripatidae – Onychophora**

>ESPP-1
MNSFIKSLCFLCVLGYAASEFECYPGALTFDDEFCNRCFCTKNGLLACTLMYCFGVRVSVSEIASAAASLPPDHF
YEAEAGNIIIAQMEDYTAAQQLAKTLEKNAGSDTVTRKSEFVSYCLKSGGNLATENLCKEIVYNGFCHIRNCFKH

Crustacea – Arthropoda - Metazoa

***Calanus finmarchicus* – Calanidae – Calanoida – Maxillipoda – Crustaeca**

>CFPP-1
MKTLVLLSGLVLSAWAAPQVTFGANDEAVPSCGLVNGEEKYVGDTWADDCNKCICRETGIATCTKRFCGNIPVIR
NPTESVVSCGLLNGEERNVGDTWADDCNNCICRETGIATCTKRFCGNIPVLKPGCTDS
>CFPP-2
MKSLFCLSVLIVWASAFPQAENGCLDSLGNTRQEGEKWEESADM*

***Gammarus pulex* – Gammaridae – Peracarida – Malacostraca – Crustacea**

>GPPP-1
PRPGALESAPVVAVALGGGRGRSADQLSDHMQCRPGSRFRQKCNWCTCSSQGLKLCSKKMCRPGADMSSEPWCEG
DAMFKDEENCNWCSCHNGVAACTLRLCISGGHRARALSASPQEPQPNTENAECVEGTSWLDECNRCRCTNGLKIC
TRRMCFSETAVTPEASEPQSDQDRCQLPVDSGPCFASFRVFRFNSETNRCEAFLYGGCQ

***Litopenaeus vannamei* – Penaeidae – Decapoda – Malacostrace – Crustacea**

>LVPP-1

MFRMKSLCVLLLFGVLVAVVESSALLGVQRTVSLPPRFTCAPGSRWKDDCNTCTCSDTGISACTLMACFGDAQQS
SDESVCDEGSRWKIDECNWCKCIRGSPACSSRPCDDHQPLTRVDGGDDIPPGGICKPGSAWKPDCNWCTCTQDGM
SASCNLRACLPDYVQDPREKVCENGSKWKLDCNTCVCVDERARCTRKACDVSRTTELPPDAVCKPGSFWKEGLQH
V

***Homarus americanus* – Nephropidae– Decapoda – Malacostraca –Crustacea**
>HAPP-1
MKVMLVLAAVLASCLAIPLEDPVAGLSEGANCVPGSRWKNDCNWCSCTETGIGMCTLMACVPEFNVEPEELVCEG
DARWKFDHCNWCSCVNGTGVCSKKICEQECKGDPNTSRWRVECNWCRCISGYGVCTRKGCPKVIMDRLDNTNECE
GTPEWKKDCNTCNCVSGRAVCTTKYCGASTDTPNTTGIEVNQEERTCT

***Carcinus maenas* – Portunidae - Decapoda – Malacostraca –Crustacea**
>CMPP-1
MKGHLLLLLLWVAISGVVCGLPDGPDAPECEGQSRNDRWRKDCNWCSCRDGKARCSRRLCAEGQKDAEPPCEGNP
AWKDDCNSCRCVDGRAVCTSKFCGQLGPQVETTHTVEVQSVQGECENGSRWRVECNWCSCQGGKSHCTEAACVNW
NESQAEEQGILECHGASRWKKDCNWCSCVNGRGRCTKRGCIPAERDSFHXLPEYAQCVPGSRWLVDCNWCSCADN
GLYSACTLMACVP

***Petrolisthes cinctipes* – Porcellanidae - Decapoda – Malacostraca –Crustacea**
>PCPP-1
MKPTPVILLVLMAVLGMAAAQGSCVPGTSWKQDCNTCFCTETGVGVCTLKLCATRGKRETPCERGSTWMDDCNRC
RCANGVGVCTKKACSVVLHDVAEAEAECQGEEGRWREACNWCRCVEGKGVCTRRGCPSDLTLFDGTPDCEGTASW
KDNCNTCHCSDGRAVCTAKLCLSNDQRIQ

Pacifastacus leniusculus – Astacidae - Decapoda – Malacostraca –Crustacea
>PLPP-1
MKALLILVMTVAAHGASLEQPDPTPASDLPDKSLCAPGSRWKNECNWCSCADHGLALCTLMGCFPGYKAAQGESV
CSEGSRWKADDCNWCRCIDGSPSCTKRLCRTKLAKGMFASQTEETECYGDPDTNRWRIECNWCRCVNGKGSCTRK
GCPQVINGIGLANTNECEGTPTWTKGCNTCSCVNGSAQCTTEECDKLVQSPSVPAVAFRSGGRTGKCRPDAHDDS
LPDYGQCVPGSRWKKDCNWCSCTETAIGMCTLIGCLNYEPKPGEAVCTDGSKWKDDCNWCTCNNGSASCTEKLCQ
YKPDGSLPDNDMCVPGSRWKDECNWCWCEANGAAPCTRMGCSEDYKPQPGEAVCIDGSRWKVDCNWCTCNNGSSA
CTEKLCLKPGGQCTEGESWRQDCNMCSCSTGLRICSVKGCPPTPT

Ellipura - Arthropoda – Metazoa

***Folsomia candida* – Isotomidae – Collembola –Ellipura**
>FCPP-1
AAQKVQQVGNSTDLETGSTANSTTTFIISEVDSSQCIDGRTKPVDCNQCVCAIGKWACTKMSCIALTLREKASYS
GDTFLNSVGTLKEHANIDGDCMEGEAKPIGCNQCVCHMNSWACSDFTCPSTPAPPKPETVQESCQTEEDVKSKDC
NICSCRRGVWVCTENDCKKFGNRYFNPVSQKHQDNEKDQES

Insecta – Arthropoda - Metazoa

Orthoptera

Gryllus bimaculatus – Gryllacrididae – Orthoptera
>GBPP-1
MTMSAVLVLLMAVALTAAHHVPGHLTDDLAHAPPVPENKCVPGTTWKLDCNTCHCTDSGISICTALGCLDNIPKL
T

Schistocerca gregaria – Acrididae - Orthoptera
>SGPP-1
MKLALALCAAFLLVVLVQAEQECTPGQTKKQDCNTCNCTPTGVWACTRKGCPPHKREVTCEPGTTFKDKCNTCRC
GSDGKSAACTLKACPQK
>SGPP-2
MAKLLAVFLVLLIAALVCEQALACTPGSRKYDGCNWCTCSSGGAWICTLKYCPPSSGGGLTFA

>SGPP-3a
MNTAASVLVLFLLALGPLVDAEKECTPGETKKLDCNTCFCSDSGIWGCTLMGCRTYTLQPAPTPGEEATRVRRSE
GHCTPNTTFKKDCNTCSCNRDGTAAVCTLKACLSRSKREVSCTPGATYKEGCNICRCRSDGKSGACTRKICPVDS
N>SGPP-4a
MKVTLAIAAAVLFVAMATTVDAASECTPGDTKKEDCNTCRCTPTGVWVCTRKGCVTKREVNCTPGATFKNKCNTC
RCGSNGRSASCTLMACPPGSY
>SGPP-5
MKVTPAIAAMALFVIMATTVAAASGCTPGEKKKEDCNTCTCTATGVWACTRRGCITKREAEESPIVKREAQKCTP
NSTFKKDCNTCTCNSSGTSAICTQLGCLSRGRRQVNCTPGTTFKDKCNTCRCSSNGRSAACTLKACPPGSY

Locusta migratoria – Acrididae – Orthoptera
>LMPP-1
MKFALALCAAVLLVVLVQAEEKCTPGQVKQQDCNTCTCTPTGVWGCTRKGCQPAKREISCEPGKTFKDKCNTCRC
GADGKSAACTLKACPNQ
>LMPP-2
MKVTLAIAAAALLVVMATTVDAAGECTPGQTKKQDCNTCTCTPTGIWGCTRKACRTTREAEEPAIVKRQAQQCTP
NKSFKKDCNTCTCNKDGTAAICTQIACLNRGRRQVNCTPGTTFQDRCNTCRCSSNGRSAACTLKACPGFG
>LMPP-3
MNVAVSVLALLLVAVGCSAEFEKECTPGETKKLDCNTCFCTKAGIWGCTLMACRTINIELTPGQNATRVRRSEEQ
CTPGTTFKKDCNTCSCGNDGTAAVCTLKACRELTTDQAGSRARRSASHCTPNTTFQKDCNTCTCNKDGTAAVCTL
KACLKRSTREVSCTPGATYKEDCNICRCRSDGKSGACTKKSCPVVED

Blattaria

Blatella germanica – Blattellinae – Blattaria
>BGPP-1
MVRLQIVTVTLLAVIATVLADCVPNSTFRQDCNTCHCSADGKTAACTQKGCVHLPLPLERSKRETCTPGSTFKRD
CNTCRCSNDGTTAACTLKACPPPPQSRRKRETESCVPGSTFKQDCNTCTCSADGRSAACTLKLCLGRQKRETCTP
GTTFKRDCNTCRCSADGQSAACTLKSCLTPGTLQA

Isoptera

Hodotermopsis sjoestedti – Termopsinae – Isoptera
>HSPP-1
MSRFFILVLLVAAAASAYAEISCEPGSVFKKDCNTCRCSDNGHAAACTRNICKPSPRQKRDTCIPGTNWKQDCNT
CSCTSSGVPACTLKACFNRPPRETPYTCTPGTNWKQDCNTCSCTSSGVPACTLKACFNRPPRETPSAQTCEPGSV
FKKECNTCTCSADGRSAACTEKACLPQNKRKRDVQKCVPGTTYKKDCNTCRCSADGQSEACTLKFCVPNPGTSVF
YSEP

Coleoptera

Tribolium castaneum – Tenebrionidae – Coleoptera
>TCPP-1a
*MKTLILCCLVLSVLIASVVS*EEAECNNGDTKKVDCNSCRCTNGLWSCTKKVCLERKTRNAFSCKPGETFKRDCNS
CTCTLDGKNAVCTLKSCVGPKA
>TCPP-1b
*MKTLILCCLVLSVLIASVVS*EEAECNNGDTKKVDCNSCRCTNGLWSCTKKVCLERKTRNTVCQPGTTFKKDCNTC
VCNKDGTNAACTLKACL
>TCPP-2a
MNSFVWVLLLVGVAVASASNSDSEVSDPHTEQCKVGDTKFKDCNFCKCTNGAFECTEKKCPDRGKRDDFS
CTPGQTFKKDCNTCTCTPDGKNAVCTLKKCAEAVANATRPN
>TCPP-2b
MNSFVWVLLLVGVAVASASNSDSEVSDPHTEQCKVGDTKFKDCNFCKCTNGAFECTEKKCPDRGKRGVPVAADLK
NTPCAPNDYFKIDCNTCYCNIEKTGYLCTENLCPLTEPPATDAPPNNATIALNDTVTESTVANFFNTTESISNHS
DTGGGTTPSLNVTL

Dascillus cervinus – Hydrophiloidae –Coleoptera
>DCPP-1

MNILKLITSIFLVILGSKFIDAAEQQSCTPGTTWKNDCNTCSCGPNGAPFCTFKACIGKREAFTPKNVGSDGTSS
ASACEPGTTWKQDCNSCSCLNGSPVCTLKACINLRKREAEQCSQGEQKNDDCNTCRCSQGQWACTKKKCNTKREA
EVCSQGQTKNEDCNTCRCANGKWACTKKKCIEKREAEDCSQGQTKNDGCNTCRCVQGQWACTRKKCNTXRETQXC
IQGDTKQEVCTLVSV

Georissus sp. – Colymbetinae – Coleoptera
>GSPP-1
MKWFVFLFVVVSIIGIALADEVKAKSGERCEQNGLTGKDDCNNCRCVDNKWACSRKLCPPVNRQKREPQRNQQCT
PGSVVKRDCNTCTCTPDGRIGACTLRACLP

Meladema coriacea - Colymbetinae – Coleoptera
>MCPP-1
PGFSCTPGKSFLNSDGCNTCTCAKDGVNAYCTLMACQSHHLDKRQSGDDKNCEPGTIIQRDCNSCNCVPGIGYAC
TKRACIKMPMQIIQKRDVGY

Cicindela campestris – Cicindelidae – Coleoptera
>CCPP-1
AACTYMACPRNKRQAEQCSPGQTKMQDCNSCRCVNGGWACTRKACPHTLQKRGNN

Pyrocoelia rufa – Lampyridae – Coleoptera
>PRPP-1
ARGQTEATICAPLSSFKIDCDDCQCSEDGTQYSCQVGVCGSAVYLNKNAQDDNSERQSRDADIPYGNDEIPPPGE
NIVADGIDSDDNSNSDSEDRGYNFYVVDKENDAEPNYANADEKRSVDDYEDAPIPYRLRRSYVE

Agriotes lineatus – Elateridae – Coleoptera
>ALPP-1
MKLLLLATVLLCVSAQNYRKCTSKFACTPGQSLTINNCQTCGCASDGQGLGICTLIECKLLQ

Diabrotica virgifera virgifera – Chrysomelidae – Coleoptera
>DVPP-1
MSRLVILLIVTVFVEYSLGGTDDSACPPLVKFRKGCNICICSPNGYDYTCTQNKCHPTKRNDNLERLLSDEDYYK
DDMSNAIDSESTTESESGTENEITDGKDVRSRHNNEFESNDMSDDDDDKDDDPDFDPDSFYDDDDDDDEDDSEDD
DHNAGKKRPASRRKYKNSKSTSDSSEIDEDESDEEY

Mecoptera

Panorpa vulgaris – Panorpidae
>PVPP-1
MNKFYVIFVFGVVCVTAASAAGGYPQGPCEPNTITKYECNDCRCAKDGSGYMCTRQVCXGPPRQKREGYPQGPCQ
PGSKTKYECNDCTCDQSGTSYMCTRRGCGFTVDSPRNKRATGYPQGPCEPNSQTRYECNSCRCTADGSGYQCTRR
ACSPAENRQKRQAEVNTPGFSCTPGSHFKHQCNDCTCMANGQFAACTLKACPPGSY

Siphonaptera

Ctenocephalides felis - Pulicidae
>CFPP-1
MKSLLLFVAVFAAAAHALPTAEPTTTAVPCTPGETKQEDCNECICKADGTGYQCTERECKHDPESKADDHGKICE
PGSTKKEDCNTCTCTPDGKNYMCTLMMCGHHHEKRETEIEEVKEVTIQSLALPMSLAKTRLDXILQCAGXDRSLT
AS
>CFPP-2
PGQETRLDCNTCKCASDGTGYFCTRQACAPVHHHKRSAEEVKEVTTTXLATTPCTPGEKTQIDCNTCTXARDXSG
YACTRKMCLPATHDRRRREAETEEVKEVTTDTLATTPCKAGEQRQVDCNTCTCAADGTGYQCTRQACEFA

Diptera

Culex quinquefasciatus – Culicidae - Diptera
>CPPP-1
MVECNKCRCSSDGKLMSCTRKFCVPDSFQSDDPAPAVAQLPTIAVDDASVASKGDEEQVHTNGQVCTPNETKQED
CNRCKCAANGIGWFCTRKACPPREKRHASRQNPLQCTPGTSFKSSDGCNDCFCTETGIAACTMKFCFNDVAKVKR

EAPKLAKQCEPGSSFKSADGCNDCFCTETGIAACTMKFCFNNKVKREAPAGTQCVKGTSFRSSDDCNTCFCGENG
VIACTRKFCVPKVKRDVQQQCVPGSTFKDAEGCNDCFCTADGRAACTEKLCLKPQKTKRDAPQPEKQCEPGTSFK
SADGCNNCFCTENGIAACTQKFCFPTKTKRQVAIGQAVPKVDCKPGTSFKHSDGCNNCYCGENGIAACTQMFCFT
KEKRDVDELPQSKLAPGTEGFECKPNSRFKYQCNQCRCDNTGKFAACTYKFCIEGEY

Armigeres subalbatus – Culicidae – Diptera

>ASPP-1
MPSQKRSLKRRRSRCTQSGQVCTPNEVKMEDCNRCKCAANGIGWFCTRKACPPRERRSASKPASQCVPGTSFKAA
DGCNNCFCGPNGIAACTQMFCFNTKTKRDTAVQPSKQQCVPGTSYKSADGCNDCFCTENGIAACTLKFCFNPRTK
RDTAVQPSKQQCVPGTSYKSADGXNDCFCTENGIAACTXKFCFNSKTKRDTAVQPPKQECVPGTSFKSADGCNDC
FCSDNGIAACTLKFCFNTKIRRDTEAVKPAERECEPGTTFRDADDCNDCFCTDDGVAACTEKFCFSEPPVIKEKR
QVKDELPVSDVAPGAPGFSCNPGKSFKFQCNTCRCDTSGQTAACTFKFCIPGEY
>ASPP-2
MRSLLLIAAVLVTGALSLGVGQQCAPGSTFKLECNTCRCSADGKLMSCTRKFCLPDEQSDD

Aedes aegypti – Culicidae – Diptera

>AAPP-1
MRSLLLIAAVLVAGALSLEAGKQCEPGSTFKLDCNTCRCGADGKVMSCTRKFCVPDVQGDDPKAQPMVDEVSDXL
QPDASAAAAAEKEEEEVHTNGQVCTPNEVKMEDCNRCKCAANGIGWFCTRKACPPRERRSASEFLTKPKRQVAQP
APPKKECEPGTNFKSADGCNDCFCTATGIAACTQKFCFNTKTKRDTAALAPEQECVPGSTFRSADDCNTCFCTET
GIAACTQKFCYDPKTFFNTKTKRDIAAQPPKKECVPGTTFKSADGCNDCFCSESGHAACTMKFCFPTPPRTKRLA
SNSKDTQCVPGTSFKSADGCNDCFCSANGMAACTLRFCFFEDQPQIKSKRQANNDELPVSAVAPGAPGFSCSPGK
SFKYQCNTCRCDTSGQTAACTFKFCLPGEY

Anopheles gambiae – Culicidae – Diptera

>AGPP-1
MKILMLIVATCVVGALCEEKCEPGTTFMEDCNKCRCGPDGQKACTRKMCPPNELSDDSQVRLDVQNGESLSSADE
KDEIHVQTNGQVCSPNEIKMKDCNRCRCANNGIGWFCTRRACPQRAKRSEPAPEKKCTPGTTFQSDDGCNTCFCT
ETGHAACTLKACLPPGFFDQQKLKQKRSVPADDLPQSAIAPGAPGFSCTPRSSFKYQCNTCLCSDDGKMAGCTFK
FCVPGEW

Belgica antarctica – Chironomidae – Diptera

>BAPP-1
KTKCIPRVPARAAARAGTTMHCVEIPHFPDTAKPAQTVQIRSDNCENGESYFDGCNTCTCKNGAYSCTLKACYTG
PLLPDTATEAVEAESTTLHPGCDKGQSYFDGCNTCVCGNGVYACTLKACFNGPLLLDTHPAPEQDKATIHNHSDG
CDKGASYNDGCNTCTCSNGNYS

Phlebotomus papatasi – Psychododae – Diptera

>PPPP-1
MKYVIFSLLLCGVSLSMGQRLENDPDGKACVPGSSFMDKDNCNRCVCTKDGARYACTKRFCGTMDIQGDQPQNEQ
NCVPGSRFKSSDGCNWCTCSEDGKHSFCTLMACVGKPEYVATPYRRKRDEGCVPKSSFTAPDGCNTCRCSDDGKH
SFCTRMECPPKAQEATPYRRRRDLTCTPGQTFTASDGCNTCHCNEEGTNAVCTLKACISNPNNPNYAATTYRRKR
DEGCTPKSTFTAPDGCNTCRCSDDGKTSFCTRMECPPTPQEATPYRRKRDLKCTPWECLYG

Lutzomyia longipalpis – Psychododae – Diptera

>LLPP-1
MKFLILTILILGVSLSMGQILENDPQGRACVPRSTFMDKEGCNRCTCTNDGARYACTKKFCTSLDMQGDEPQRCT
PGSTFKAADGCNTCRCSDDGKNSMCTLMACIGNPTAENAPLPYRRKRDDSECVPKSTFTAADGCNTCTCSENGKY
AFCTRMECPPKQVATPYRRRRDLGN

Lepidoptera

Spodoptera frugiperda – Noctuidae – Lepidoptera

>SFPP-1
MKWSIFVCLLVCLASYCDGLAIKCSPGVIDPSCTEEKKEVDRHESQPRDPTEVQCLVGSEWESNCHYCRCSDEGV
AECLRQDTCDKGVFAEPVLCKPNTTFQRDCNTCICLENGLGLCTLEFCRRSGSIDSFPEILIADTELNSKDEASQ
PFKTEIVVPTTKSAKNKTKSRVCEANRMFIKDCNTCWCNEDGTSYYCTRRVCVPLLPEDQPDIGGIPAEIFA

Bombyx mori – Bombycidae – Lepidoptera

>BMPP-1

MRCLIAICFIVLARHCESGALKCSPGTEGPCAAEQESKDKPSQITTDDASAVQEMQSEVECLAGTEWESNCHFCR
CSDSGVAECLRQDSCDQIIFTEPVRCQPGTSFQRDCNTCVCLDNGLGLCSLDACRRSSTPKKFELIQGRECAPGS
SWSNQCNSCRCNADGYGICSDEACTEHIIEPKKECAPKTMWKNECNTCWCTSDGKPMCTRMECITNNTPEKSELI
QGRECAPGSTWSNQCNSCRCNADGYAICSDEACAEHINEPKKDCVPNTTWKNECYTCWCTSDGKPMCTRVECITN
NTPKKSELIQGRDCAPGSTWSNQCNSCRCNADGYAICSDEACAEHIDEPKKDCAPKTMWKNECNTCWCTSDGKPM
CTKMGCISYNNFGSGVTEKLETKGTEIPELRSSQNTTKAIVCVANRMFIKDCNTCWCNEDGTTFYCTRRVCVPML
PEDGDEDPENLNQKPATPKECKPNETFQIGCNRCRCNSEGTLYSCTRIGCLESEEKNHTLSRKVRASQQETVKTC
QPGQEFRLDCNKCLCDKEGKDFSCTRMDCNALNSNHNAEPFNGDRTKREVSQKPATCVPGSVYNQGCNVCRCTDE
GRHATCTLMRCPQEKEETHAHDQDPGFRCNPGEQFTRDCNDCTCSADGKSVFCTLRLCDQDITPHINA

Antheraea mylitta – Saturniidae - Lepidoptera

>AMPP-1
MRCLLIFGLFVLAVHGAESSLRSAVRSLKTCEPGTHWKEGCNFCYCVEAGIPACTTLLCHGLEKIRDLPELQLPS
GVDCLPGTTWKSQCNYCLCLGDGYPACTFKECPWNSHEPEKTCAAHTIWRDECIVCRCTIYGEASCSMLACNYPL
DEDLISKLKNEL

Samia cynthia ricini – Saturniidae - Lepidoptera

>SCPP-1
SNVDAECLPRSKWESNCHSCECSESGAAKCVKQKDCLDEHVLAEPIRCKPNTTFNKGCNTCLCLENGLGLCTLQN
CWRSSIPKKSELLPGKDCAPGSSWRSQCNDCVCNDQGISLCTLALCPDQEQEPIMNCAPNTMWKNDCNTCRCTTA
GKAMCTRIGCIS

Heliconius erato – Nymphalidae - Lepidoptera

>HEPP-1nostop
MKWIVIASFMVYLAAFSEGSALRCSRGTDNDCHTDNTEKEAESSVDSKKPECLAGSEWESLCHSCRCSEDGQPEC
ARLGSCRDETKSEPMVCKPLTEFRRDCNICICFESGLGSSCTSMECAPKTDISNGKECKVGTKWQSQCNNCSCSD
DGMPMCTDMACPGQEEEPEMLCALGTSWKLDCNTCYCPPTGRAACTRIGC
>HEPP-2 no signal yes stop
QKRKKRKRTEVCGIAPREENEDETESLQEIKRKCRPDEVFEVDCNMCRCSVDGMSFSCTRRACVPEDDGKDASLL
RKARATSQGTRKACQPGQEFTMDCNKCLCNNEGQDYSCTRINCAELNSNGNNGARAKREVATQVKADCVPGSVFK
QGCNTCQCTEDGNHATCTIKRCKEDNPDENEVNLPESDPSFRCNPGEQFKRGCNDCACSADGKSVFCALRLCDQD
ITLTI

Danaus plexippus – Nymphalidae - Lepidoptera

>DPPP-1
QEFRMDCNKCLCDNEGQNFSCTRIDCAALNSNGNGGTRVRREVSTREESGCTPGSVFTQDCNTCRCTEDGGHATC
TLKQCVKHDTGYELNQPESDPNFRCNPGEQFKRDCNDCTCSANGRGVFCTLRICDFEI
>DPPP-2
MKCSVVAFILCLATFCRGGVIKCTPGSKDETCVTDNFGKESLVRQKRQDSIVEECSPRTEWKSKCHRCICSDSGQ
ALCFKIEGCRSDSGEPIRCKPESKFSRDCNSCLCTNNGNVICTLKACLPSLVKEQARNDVSEDTRFRRSVAPSKP
VVCAANRMFIKDCNTCWCNEDGTSFFCTRKVCVEELPEEVSEPVKFMKLTARAGLMKCL

Manduca sexta – Sphingidae - Lepidoptera

MSPP-1
MMWLLFAALLVFARHADCGALRCPEGSSCTAEEESKAPPLPVPNNPSPREAECQPGSEWESNCHSCKCSEAGIAE
CLKQAACDKEISADPIRCKPNTNVAEGLPHLHLSGRRIRLCTLKNCAKNHPELPSAKTAPQETSWMNECHHCWCT
SKGYRACTLKGCFHDPEPTILRSQHFLEEKCYICPLFYSGAAFCPLMECRENTRPSLAEIYEDNSEHEQRSQLKP
LTDIKVEKRATRCEPRAEFKSECNVCKCSADGRSFSCTQNECLEGDSVDSDVEVFQETDGPDHVERNSVCQPNTI
FYVACNACQCNNQGTDFACTLKICPLPRDVEVFHEFRDMSPVVPMMGVSDAG

Epiphyas postvittana – Tortricidae - Lepidoptera

>EPPP-1
NSCTCLENGLGLCTLKNCHIIPARAPSKEDLRMGQDCAPGSEWKSGCNDCVCTPEGLRSCSSAGCSEAQESEPLL
RCALHSLWNKESNTCWCTSDGRAMCTKIGCVGIPSDMHFSDEEDESGTQEVVESGTISKVPKDADSDEPENTDWN
SDIDDADIELDEDNVTGLALRDLEDFDDN

Hymenoptera

Apis melifera – Apidae - Hymenoptera

>AMPP-1

MSFKSFLILYAIAIDLAIQKPMMCVPGKSFFDGCNTCTCTDDGNFICTMTACEDYDPETDTSVPVKILEPPPDFW
QNS

Nasonia vitripennis – Pteromalidae - Hymenoptera

>NVPP-1
MGESILILTIITLGIVARANCITFRDPTPTLECVPNRLFTYDHNICYCNRDGTELTCKRKMYSTLTPDLYLVQLQ
NLTMECIPKRHFQFSNQDCICHESGRFASCVRKNQDNGLDSRENCFPGAVFQDDCNGCICGSDGKATCTNMDCNM
LDNINSDGKPKPSDLQCVPGSELIHRCNQCFCTDSGTAMMCFKMGCGALSLIHEHVLNVTMNCQADKIFDYNCHQ
CICDAKGNYAMCSGKECPRSDVFKEVKDTVEKCNPGMIFASDCNVCICSKNGKGVCTTFSCDTTYRFKYFDQLSL
QKPRMTLHDRLIFV
>NVPP-2
MKSQFVVLLAIVSAASARNIFSCLPGSVFLQDCNACTCSNDGLSAACTDMACPGDLNRLTVFQPVLLQPAKVCEP
STVFKVYCNTCGCSSDGSSFSCTRMACNQDIWNVDGSLKFQSTAVRTKRSLAPQEKVCEPRTQFKEYCNTCGCAD
DGLSYICTRRMCDENIWNKDGSLKIDITKDVVKRSAPKQICKPHSNFKDYCNTCFCNNDGSEFACTRMSCPPEVW
NKDGSLKIRDVRLEKREAKQVCEPRSHFKDYCNTCACSEDGTTYGCTMMMCDESVWNKDGTRKTVDGETN
>NVPP-3
MRSFLCFVTFLALVLAVSASPIDIKRNDDFGSTGKCTPGQVFFMSCNLCKCSSDGNYAACTFMQCFDFNFEEEQR
SKRSTNEVVAKLSSDIPRISGYTQGDQCPSKSFYNDCNMCVCGPDDASAACTMMMCMPGETQQPSKIVPAKLNDI
ARIDEYSQGQACPAGEFFHDKCNVCHCSANGFSAACTLMGCPSEDTTQPRAQVTEIYNWPRIGNNHRANIC
>NVPP-4
MKTLLIFVVSTIALVSSEFHCTPGSTFQMDCNSCTCSNDGKTAMCTGIACIQENKSDVTDPGEKGENLAPIQTQG
VGQAEFHCTPGSNFHQDCNSCICLKDGQSAMCTGIACPTKVKRDLETGPQQVCVPKSKFNDYCNTCGCSDDGSSF
ICTRRLCDPEVWNKDGTMKISPKSLQRAARSISPEHKCKPRHLFKKDCNHCVCNAGGETAQCTVLDCSKLDLSQF
HNNPLYIKPAQNPRK
>NVPP-5
MASKTTCLLLLGLLIIATLYVSVAEAKKQCVPGKSYFDGCNTCFCSEAHSVQCTRRLCPDPWKRLSPPADFYQ
>NVPP-6
MSKVLKVGLLLLLVAVAASAYAVAEENGAPKENKQLPQIDDYGVTNKCPANQPFKWNCNYCTCGPEGKDASCTRM
ACPQH
>NVPP-7
MHSSTLIVVINSIFLYFCTDNANDLPHIDNYGETNECPPNESFMDKCNYCRCGPEGKDAACTKMNCP
>NVPP-8a
MRSLFIILVAVAVASANQVCTPGTYFKTECNTCVCAKDGSASICTQKQCPPGLFNWDGSLKVPLDIIQQIIPDVS
QIEIFESQLVAVGEVTYVQTCTPNSFFHINCNRCQCSKHGIQYECEKNACPVQPHF
>NVPP-8b
MRSLFIILVAVAVASANQVCTPGTYFKTECNTCVCAKDGSASICTQKQCPPGLFNWDGSLKVPLDIIQQIIPDVS
QIEIFESLKKVCEPNQVFQNNCNTCACNKDSTAAACTLKECN

Nasonia Giraulti – Pteromalidae - Hymenoptera

>NGPP-1 (NVPP-5)
MASKTTCLLLLGLLIIATLYVSVAEAKKQCVPGKSYSDGCNTCFCSEAHSVQCTRRLCPDPWKPLSPPADFYQ
>NGPP-2 (NVPP-6)
MSKVLKVGLLLLLVAVATSAYAVAEENAAPEDSVVEVEAEKGTDNQTQNKQLPQIDDYGVTNKCPANQSFKWNCN
YCTCGPEGKDASCTRMACP
>NGPP-3 (NVPP-7)
MHSSTLIVVINSIFLYFCIDDANNLPHIDNYGETNECPPNESFIHKCNYCKCGPEGKDAACTKMNCP
>NGPP-4
MYFTSTFSLLMLAIFSVSYADDVEMQCKPKTRFKFYCNTCWCSEEGTTRICTKKYCPDNIFNKDGSLKTPPNVHQ
KLKILPKGVGSPD
>NGPP-5
MYFTSTFSLLMLAIFSVSYADDVEMQCKPKTRFKFYCNTCWCSEEGTTRICTKKYCPDNIFNKDGSLKTPPNVHQ
KLKILPKGVGSPD

Pimpla hypochondriaca – Ichneumonidae - Hymenoptera

>PHPP-1
MGFLACALLVVATAHAATAIVNPETCEIGSNFKNYCNNCYCFDGVMDHALCTRESCDRNVWNEDGTRKFPKPGKW
ISEKENKKNDEPCTPGENFKYYCNDCQCLDGLRAHAMCTRMRCDRNVFNEDGTRKYPEPEKWNSEKERRKKSDESC
APGASFKYYCNSCTCGAEGKVAEAQCTSQECDRYKWKKDGSKRPFTLDPVLHD

Solenopsis invicta – Formicidae - Hymenoptera

>SIPP-1
AGPYQYFSFRGPSSVCIPGSSYFDGCNNCFCQNDAHTVGCTTNWCMYATKIPPPDDFWQ

Hemiptera

Myzus persicae - Aphididae – Hemiptera
>MPPP-1
MRAFCDCACDESPGPFKMRIGGRTYYQLQFLIVVFSAVAATSVHGLRNVRPDGPCDPGELVFVGFCNLCLCNSQG
IPNQLCARSWCPVQTTSPPRNGILYSNTNKPFT

Acyrthosiphon pisum – Aphididae - Hemiptera
>APPP-1
MRVEGRTFCQLQLLLIVVFSAATSVHCLRNVKPDGPCDPGELVFVGFCNLCMCNSQGMPNQLCARSWCPVQTTLP
PRNSILYSIPNKPFT

Nilaparvata lugens – Delphacidae - Hemiptera
>NLPP-1
MAFTKQWKGRGLIVCMVLISIFQMASPYKISRDVETCLPGELVWVKCNLCLCNLEGQPNAVCAKMWCQPTPKYHY
DGDDVDTREIVN

Rhodnius prolixus – Reduviidae - Hemiptera
>RPPP-1
GRPGQVLIMIVLITNGRSKNLRTRRDTGEKKCKPGSVWKEDCNTCFCTETGHVGCTLMHCGPISSQQTRVKREKQ
CTPGTTWKEDCNTCFCSSTGQIGCTLMACHHYQLPTKQQRFKRDFEDEEQSEVSRQEETGKNCEI
GTTVKLDCNICHGTSAATTLI

Triatoma infestans – Reduviidae - Hemiptera
>TIPP-1
MKTSAVFLFLVATMAIGTTALYCEPNTRFKQECNWCTCSANGEYATCTLLYCGPRRARAVSSGIYCGFGGTVPAG
DGCNFCFCTPLGTIGTCTMRRCDSLS

Oncopeltus fasciatus – Lygaeinae - Hemiptera
>OFPP-1
SRARTVCCVAGCTPGSSWREKCKSCVCSKNGTPSCTRILCTGQGGLDLQLPDLKESADEESELNDSLRICYF

Oncometopia nigricans – Cicadellidae - Hemiptera
>ONPP-1
MNNKAMFGLASLAFLLLSSVKAGASELTTASGIGSYDNCVPGTTWMDDCNTCHCNVNGLAACTRMLCFPGGIPKH
CTPGSSWTENCQTCTCSDQGAVTCTGDSCSTPPTPSVI

Graphocephala atropunctata – Cicadellidae - Hemiptera
>GAPP-1
MKSLLVVALGVFLIGSIMADTSAESPESPEIVQECVPGTTWKNDCNSCFCSETGVAACTLMGCIPHNIQVPSTCT
PGSTWTDNCQSCTCSEEGKASCTGAPCTLLST

Phthiraptera

Pediculus humanus – Pediculidae – Phthiraptera
>PHPP-1
MKYALVFAFFAVFAIASAFPSENSEKEEETKSDLKKLDDAKVEETGAQAKSSVSDEERCEPGQSFAKECNTCTCP
DSGLKSLAGCTLKLCL
>PHPP-2
MKFQMILMAALSCFLIMEIQAKPSNVCEPGSTFKMECNSCKCTDDGTDMVCTKKLCVPENGGEHKEGEHKEQPQG
DQPKEETQSVTDDEHKDKPQSDQAAEETHAETEEHKEQTEDGKPKEGGDDAAKTETHEDEKKEETEEKPHTETET
KTEEKTKEAEHTEEHVQNDEAKAEDVEDKKEETEEKKEEAEEKKEEKAEDKDEDSCTPGETFKKLCNDCTCPPTG
HKSAATCTLLTCGEEAH
