# Supplementary material for: Identification, distribution and molecular evolution of the pacifastin gene family in Metazoa
Source: BMC Evol Biol. 2009 May 12;9:97. doi: 10.1186/1471-2148-9-97 (PMC2689174; doi:10.1186/1471-2148-9-97)
Supplement: Additional file 4 — List of all previously and newly identified PLD-related inhibitor domains in Metazoa. The amino acid sequences of PLD-like domains are given in FASTA format and categorized according to the species' classification (family, order, phylum and regnum). The conserved cysteine residues are highlighted in grey, while the catalytic residues of the reactive site (P1-P1') are highlighted in black. [file 1471-2148-9-97-S4.pdf]

Placozoa - Metazoa

Trichplax adhaerens

>TAPD-1
CKFNNINYQVGENRKAEDGCNTCMCLKNGGWGCTRMAC
>TAPD-2
CIFEEKLFRLGETVKQKCNQCKCLGQNKWACTRIAC
>TAPD-3
CVHNTMLLKQNETKRKDCNTCKCLGSNRWACTRMFC
>TAPD-4
CIFEGKMFRKEETRADDCNICRCFGNNRWGCTRKAC
>TAPD-5
CNYGGRRFRTGDYVARGCNVCRCTPTGKFMCTRNDC
>TAPD-6
CILNDREYNISAVTKIDCNYCRCLKGIWACTRMAC
>TAPD-7
CEKDGRFFVRGELRREKCNLCRCSNSGRWMCTTKMC
>TAPD-8
CQHKTRTFFHGEIRKDQCNFCNCTRNETWACSNYSC
>TAPD-9
CVHRGRRFNIGHIKKQSCNTCKCLTNGGWGCTRMMC
>TAPD-10
CQMGNEVSRVGETKFLDCNKCTCSSTGKWMCTEKVC
>TAPD-11
CKFNNQYFQKGEQIRNSCNLCKCGYTGQWACTRMMC
>TAPD-12
CTYQNTTLYLGEVRKFNCSYCSCRGNETVECMELPC
>TAPD-13
CYHMGTTYPTGFIAKKDSCNKCKCNKYGLWACTAFKC
>TAPD-14
CTHNNEIYRPSEIRMDDCNRCMCLKDGAWGCTRMMC
>TAPD-15
CTFRGRTFAAGTIKKQGCNMCKCYTNGEWNCTTKPC
>TAPD-16
CEMRGMFLFHGETIRKNCNLCTCLPTGNMACTAKDC
>TAPD-17
CKHGKRLFYEGERSKQDCNECQCLSNGSWNCTQNTC
>TAPD-18
CRMMDRTFEVGHRMRHRCRHCRCMEGGFWGCRHRPC
>TAPD-19
CRHSGLKFNHGENRSLDCNTCICNNGSWACTMKMC
>TAPD-20
CVHNNEYYSHRETRKQDCNTCMCTNGKWACTRMMC
>TAPD-21
CFHENETFQHGATRKQDCNTCKCIDGRWGCTRMMC
>TAPD-22
CQYKDEMFQQGEITKQDCNTCKCIKGRWACTRMLC
>TAPD-23
CEYEDTIYEHNETRKQDCNTCKCITGKWACTRMMC
>TAPD-24
CTFRDTLYEHGETRKQDCNTCKCITGKWACTRMMC
>TAPD-25
CQYKDTTFAHGETTKQDCNTCKCIKGRWACTRMLC
>TAPD-26
CEYEDTIYEHNETRKQDCNTCKCITGKWACTRMMC
>TAPD-27
CTFRDTLYEHGETRKQDCNTCKCITGKWACTRMMC
>TAPD-28
CQYKDTTFAHGETTKQDCNTCKCIKGRWACTRMLC
>TAPD-29

CEYEDTIYEHNETRKQDCNTCKCITGKWACTRMMC
>TAPD-30
CTYRDTLYEHGETRKQDCNTCKCITGKWACTRMMC
>TAPD-31
CQYKDTTFAHGETTKQDCNTCKCIKGRWACTRMLC
>TAPD-32
CEYEDTIYEHNETRKQDCNTCKCITGKWACTRMMC
>TAPD-33
CTYRDTLYEHGETRKQDCNTCKCITGKWACTRMMC
>TAPD-34
CQYKDTTFAHGETTKQDCNTCKCIKGRWACTRMLCV
>TAPD-35
CEYEDTIYEHNETRKQDCNTCKCITGKWACTRMMC
>TAPD-36
CQYKDTTFAHGETTKQDCNTCKCIKGRWACTRMLC
>TAPD-37
CEYEDTIYEHNETRKQDCNTCKCITGKWACTRMMC
>TAPD-38
CTYRDTLYEHGETRKQDCNTCKCITGKWACTRMMC
>TAPD-39
CVYEKKNYNITAVVEKDCQRCICGLFARWYCQRKPNC
>TAPD-40
CALGSQQVPTGYKMEEKCRQCTCMNDGQWKCNVNSEC
>TAPD-41
CTLTNGQIIPHGHVEEKKCEKCKCNDGKYVCTNKPC
>TAPD-42
CEYQNKKFPVNTVKLIGLCHRCKCSPAGNWRCLDLGC

Onychophora -Metazoa

***Epiperipatus sp.* – Peripatidae – Onychophora – Metazoa**
>ESPD-1
CEPGTTYKNDCNTCHCNADGTAGPCTLMFC
>ESPD-2
CEPNTSFKRDCNSCHCNADGTVAACTLMTC
>ESPD-3
CYPGALTFDDEFCNRCFCTKNGLLACTLMYC

Crustacea – Arthropoda - Metazoa

***Calanus finmarchicus* – Calanidae – Calanoida – Maxillipoda – Crustaeca**
>CFPD-1
CGLVNGEEKYVGDTWADDCNKCICRETGIATCTKRFC
>CFPD-2
CGLLNGEERNVGDTWADDCNNCICRETGIATCTKRFC
>CFPD-3
CGENERGVLRQPGDSWQEDCNRCRCLAAGVPGCTKKFC

Gammarus pulex – Gammaridae – Peracarida – Malacostraca – Crustacea
>GPPD-1
CRPGSRFRQKCNWCTCSSQGLKLCSKKMC
>GPPD-2
CEGDAMFKDEENCNWCSCHNGVAACTLRLC
>GPPD-3
CVEGTSWLDECNRCRCTNGLKICTRRMC

***Litopenaeus vannamei* – Penaeidae – Decapoda – Malacostrace – Crustacea**
>LVPD-1
CAPGSRWKDDCNTCTCSDTGISACTLMAC

>LVPD-2
CDEGSRWKIDECNWCKCIRGSPACSSRPC
>LVPD-3
CKPGSAWKPDCNWCTCTQDGMSASCNLRAC
>LVPD-4
CENGSKWKLDCNTCVCVDERARCTRKAC

***Homarus americanus* – Nephropidae– Decapoda – Malacostraca –Crustacea**
>HAPD-1
CVPGSRWKNDCNWCSCTETGIGMCTLMAC
>HAPD-2
CEGDARWKFDHCNWCSCVNGTGVCSKKIC
>HAPD-3
CKGDPNTSRWRVECNWCRCISGYGVCTRKGC
>HAPD-4
CEGTPEWKKDCNTCNCVSGRAVCTTKYC

Carcinus maenas – Portunidae - Decapoda – Malacostraca –Crustacea
>CMPD-1
CEGQSRNDRWRKDCNWCSCRDGKARCSRRLC
>CMPD-2
CEGNPAWKDDCNSCRCVDGRAVCTSKFC
>CMPD-3
CENGSRWRVECNWCSCQGGKSHCTEAAC
>CMPD-4
CHGASRWKKDCNWCSCVNGRGRCTKRGC
>CMPD-5
CVPGSRWLVDCNWCSCADNGLYSACTLMAC

Petrolisthes cinctipes – Porcellanidae - Decapoda – Malacostraca –Crustacea
>PCPD-1
CVPGTSWKQDCNTCFCTETGVGVCTLKLC
>PCPD-2
CERGSTWMDDCNRCRCANGVGVCTKKAC
>PCPD-3
CQGEEGRWREACNWCRCVEGKGVCTRRGC
>PCPD-4
CEGTASWKDNCNTCHCSDGRAVCTAKLC

Pacifastacus leniusculus – Astacidae - Decapoda – Malacostraca –Crustacea
>PLPD-1
CAPGSRWKNECNWCSCADHGLALCTLMGC
>PLPD-2
CSEGSRWKADDCNWCRCIDGSPSCTKRLC
>PLPD-3
CYGDPDTNRWRIECNWCRCVNGKGSCTRKGC
>PLPD-4
CEGTPTWTKGCNTCSCVNGSAQCTTEEC
>PLPD-5
CVPGSRWKKDCNWCSCTETAIGMCTLIGC
>PLPD-6
CTDGSKWKDDCNWCTCNNGSASCTEKLC
>PLPD-7
CVPGSRWKDECNWCWCEANGAAPCTRMGC
>PLPD-8
CIDGSRWKVDCNWCTCNNGSSACTEKLC
>PLPD-9
CTEGESWRQDCNMCSCSTGLRICSVKGC

Ellipura - Arhtropoda – Metazoa

***Folsomia candida* – Isotomidae – Collembola –Ellipura**

>FCPD-1
CIDGRTKPVDCNQCVCAIGKWACTKMSC
>FCPD-2
CMEGEAKPIGCNQCVCHMNSWACSDFTC
>FCPD-3
CQTEEDVKSKDCNICSCRRGVWVCTENDC

Insecta – Arthropoda - Metazoa

Orthoptera

Gryllus bimaculatus – Gryllacrididae - Orthoptera

>GBPD-1
CVPGTTWKLDCNTCHCTDSGISICTALGC

Schistocerca gregaria – Acrididae - Orthoptera

>SGPI-1
CTPGQTKKQDCNTCNCTPTGVWACTRKGC
>SGPI-2
CEPGTTFKDKCNTCRCGSDGKSAACTLKAC
>SGPI-3
CTPGSRKYDGCNWCTCSSGGAWICTLKYC
>SGPI-4A
CTPGETKKLDCNTCFCSDSGIWGCTLMGC
>SGPI-4B
CTPNTTFKKDCNTCSCNRDGTAAVCTLKAC
>SGPI-4Ca
CTPGATYKEGCNTCRCRSDGKSGACTRKIC
>SGPI-4Cp
CTPGATYKEGCNTCRCRSDGKSGACTRKIC
>SGPI-5A
CTPGDTKKEDCNTCRCTPTGVWVCTRKGCV
>SGPI-5Ba
CTPGATFKNKCNTCRCGSNGRSASCTLMAC
>SGPI-5Bt
CTPGTTFKNKCNTCRCGSNGRSASCTLMAC
>SGPI-6
CTPGEKKKEDCNTCTCTATGVWACTRRGC
>SGPI-7
CTPNSTFKKDCNTCTCNSSGTSAICTQLGC
>SGPI-8
CTPGTTFKDKCNTCRCSSNGRSAACTLKAC

Locusta migratoria – Acrididae – Orthoptera

>LMPI-1
CTPGQVKQQDCNTCTCTPTGVWGCTRKGC
>LMPI-2
CEPGKTFKDKCNTCRCGADGKSAACTLKAC
>LMPI-3
CTPGQTKKQDCNTCTCTPTGIWGCTRKAC
>LMPI-4
CTPNKSFKKDCNTCTCNKDGTAAICTQIAC
>LMPI-5
CTPGTTFQDRCNTCRCSSNGRSAACTLKAC
>LMPI-6
CTPGETKKLDCNTCFCTKAGIWGCTLMAC
>LMPI-7
CTPGTTFKKDCNTCSCGNDGTAAVCTLKAC

>LMPI-8
CTPNTTFQKDCNTCTCNKDGTAAVCTLKAC
>LMPI-9
CTPGATYKEDCNICRCRSDGKSGACTKKSC

Blattaria

Blatella germanica – Blattellinae – Blattaria
>BGPD-1
CVPNSTFRQDCNTCHCSADGKTAACTQKGC
>BGPD-2
CTPGSTFKRDCNTCRCSNDGTTAACTLKAC
>BGPD-3
CVPGSTFKQDCNTCTCSADGRSAACTLKLC
>BGPD-4
CTPGTTFKRDCNTCRCSADGQSAACTLKSC

Isoptera

Hodotermopsis sjoestedti – Termopsinae – Isoptera
>HSPD-1
CEPGSVFKKDCNTCRCSDNGHAAACTRNIC
>HSPD-2
CIPGTNWKQDCNTCSCTSSGVPACTLKAC
>HSPD-3
CTPGTNWKQDCNTCSCTSSGVPACTLKAC
>HSPD-4
CEPGSVFKKECNTCTCSADGRSAACTEKAC
>HSPD-5
CVPGTTYKKDCNTCRCSADGQSEACTLKFC

Coleoptera

Tribolium castaneum – Tenebrionidae – Coleoptera
>TCPD-1
CKPGETFKRDCNSCTCTLDGKNAVCTLKSC
>TCPD-2
CKPGETFKRDCNSCTCTLDGKNAVCTLKSC
>TCPD-3
CQPGTTFKKDCNTCVCNKDGTNAACTLKAC
>TCPD-4
CKVGDTKFKDCNFCKCTNGAFECTEKKC
>TCPD-5
CTPGQTFKKDCNTCTCTPDGKNAVCTLKKC
>TCPD-6
CAPNDYFKIDCNTCYCNIEKTGYLCTENLC

Dascillus cervinus – Hydrophiloidae –Coleoptera
>DCPD-1
CTPGTTWKNDCNTCSCGPNGAPFCTFKAC
> DCPD-2
CEPGTTWKQDCNSCSCLNGSPVCTLKAC
>DCPD-3
CSQGEQKNDDCNTCRCSQGQWACTKKKC
>DCPD-4
CSQGQTKNEDCNTCRCANGKWACTKKKC
>DCPD-5
CSQGQTKNDGCNTCRCVQGQWACTRKKC

Georissus sp. – Colymbetinae – Coleoptera

>GSPD-1
CEQNGLTGKDDCNNCRCVDNKWACSRKLC
>GSPD-2
CTPGSVVKRDCNTCTCTPDGRIGACTLRAC

Meladema coriacea - Colymbetinae – Coleoptera

>MCPD-1
CTPGKSFLNSDGCNTCTCAKDGVNAYCTLMAC
>MCPD-2
CEPGTIIQRDCNSCNCVPGIGYACTKRAC

Cicindela campestris – Cicindelidae – Coleoptera

>CCPD-1
CSPGQTKMQDCNSCRCVNGGWACTRKAC

Pyrocoelia rufa – Lampyridae – Coleoptera

>PRPD-1
CAPLSSFKIDCDDCQCSEDGTQYSCQVGVC

Diabrotica virgifera virgifera – Chrysomelidae – Coleoptera

>DVPD-1
CPPLVKFRKGCNICICSPNGYDYTCTQNKC

Mecoptera

Panorpa vulgaris - Panorpidae

>PVPD-1
CEPNTITKYECNDCRCAKDGSGYMCTRQVC
>PVPD-2
CQPGSKTKYECNDCTCDQSGTSYMCTRRGC
>PVPD-3
CEPNSQTRYECNSCRCTADGSGYQCTRRAC
>PVPD-4
CTPGSHFKHQCNDCTCMANGQFAACTLKAC

Siphonaptera

Ctenocephalides felis - Pulicidae

>CFPD-1
CTPGETKQEDCNECICKADGTGYQCTEREC
>CFPD-2
CEPGSTKKEDCNTCTCTPDGKNYMCTLMMC
>CFPD-3
PGQETRLDCNTCKCASDGTGYFCTRQAC
>CFPD-4
CTPGEKTQIDCNTCTXARDXSGYACTRKMC
>CFDP-5
CKAGEQRQVDCNTCTCAADGTGYQCTRQAC

Diptera

Culex quinquefasciatus – Culicidae - Diptera

>CPPD-1
-------MVECNKCRCSSDGKLMSCTRKFC
>CPPD-2
CTPNETKQEDCNRCKCAANGIGWFCTRKAC
>CPPD-3
CTPGTSFKSSDGCNDCFCTETGIAACTMKFC

>CQPD-4
CEPGSSFKSADGCNDCFCTETGIAACTMKFC
>CQPD-5
CVKGTSFRSSDDCNTCFCGENGVIACTRKFC
>CQPD-6
CVPGSTFKDAEGCNDCFCTADGRAACTEKLC
>CQPD-7
CEPGTSFKSADGCNNCFCTENGIAACTQKFC
>CQPD-8
CKPGTSFKHSDGCNNCYCGENGIAACTQMFC
>CQPD-9
CKPNSRFKYQCNQCRCDNTGKFAACTYKFC

Armigeres subalbatus – Culicidae - Diptera
>ASPI-1
CTPNEVKMEDCNRCKCAANGIGWFCTRKAC
>ASPI-2
CVPGTSFKAADGCNNCFCGPNGIAA-CTQMFC
>ASPI-3
CVPGTSYKSADGCNDCFCTENGIAA-CTLKFC
>ASPI-4
CVPGTSYKSADGCNDCFCTENGIAA-CTXKFC
>ASPI-5
CVPGTSFKSADGCNDCFCSDNGIAA-CTLKFC
>ASPI-6
CEPGTTFRDADDCNDCFCTDDGVAA-CTEKFC
>ASPI-7
CNPGKSFKFQCNTCRCDTSGQTAACTFKFC
>ASPI-8
CAPGSTFKLECNTCRCSADGKLMSCTRKFCLPDEQSDD

Aedes aegypti – Culicidae - Diptera
>AAPD-1
CEPGSTFKLDCNTCRCGADGKVMSCTRKFC
>AAPD-2
CTPNEVKMEDCNRCKCAANGIGWFCTRKAC
>AAPD-3
CEPGTNFKSADGCNDCFCTATGIAACTQKFC
>AAPD-4
CVPGSTFRSADDCNTCFCTETGIAACTQKFC
>AAPD-5
CVPGTTFKSADGCNDCFCSESGHAACTMKFC
>AAPD-6
CVPGTSFKSADGCNDCFCSANGMAACTLRFC
>AAPD-7
CSPGKSFKYQCNTCRCDTSGQTAACTFKFC

Anopheles gambiae – Culicidae - Diptera
>AGPD-1
CEPGTTFMEDCNKCRCGPDGQKACTRKMC
>AGPD-2
CSPNEIKMKDCNRCRCANNGIGWFCTRRAC
>AGPD-3
CTPGTTFRSDDGCNTCFCTETGHAACTLKAC
>AGPD-4
CTPRSSFKYQCNTCLCSDDGKMAGCTFKFC

Belgica antarctica – Chironomidae - Diptera
>BAPD-1
CENGESYFDGCNTCTCKNGAYSCTLKAC
>BAPD-2

CDKGQSYFDGCNTCVCGNGVYACTLKAC
>BAPD-3
CDKGASYNDGCNTCTCSNGNYS

Phlebotomus papatasi – Psychododae - Diptera

>PPPD-1
CVPGSSFMDKDNCNRCVCTKDGARYACTKRFC
>PPPD-2
CVPGSRFKSSDGCNWCTCSEDGKHSFCTLMAC
>PPPD-3
CVPKSSFTAPDGCNTCRCSDDGKHSFCTRMEC
>PPPD-4
CTPGQTFTASDGCNTCHCNEEGTNAVCTLKAC
>PPPD-5
CTPKSTFTAPDGCNTCRCSDDGKTSFCTRMEC

Lutzomyia longipalpis – Psychododae - Diptera

>LLPD-1
CVPRSTFMDKEGCNRCTCTNDGARYACTKKFC
>LLPD-2
CTPGSTFKAADGCNTCRCSDDGKNSMCTLMAC
>LLPD-3
CVPKSTFTAADGCNTCTCSENGKYAFCTRMEC

Lepidoptera

Spodoptera frugiperda – Noctuidae - Lepidoptera

>SFPD-1
CLVGSEWESNCHYCRCSDEGVAECLRQDTC
>SFPD-2
CKPNTTFQRDCNTCICLENGLGLCTLEFC
>SFPD-3
CEANRMFIKDCNTCWCNEDGTSYYCTRRVC

Bombyx mori – Bombycidae - Lepidoptera

>BMPD-1
CLAGTEWESNCHFCRCSDSGVAECLRQDSC
>BMPD-2
CQPGTSFQRDCNTCVCLDNGLGLCSLDAC
>BMPD-3
CAPGSSWSNQCNSCRCNAFGYGICSDEAC
>BMPD-4
CAPKTMWKNECNTCWCTSDGKPMCTRMEC
>BMPD-5
CAPGSTWSNQCNSCRCNADGYAICSDEAC
>BMPD-6
CVPNTTWKNECYTCWCTSDGKPMCTRVEC
>BMPD-7
CAPGSTWSNQCNSCRCNADGYAICSDEAC
>BMPD-8
CAPKTMWKNECNTCWCTSDGKPMCTKMGC
>BMPD-9
CVANRMFIKDCNTCWCNEDGTTFYCTRRVC
>BMPD-10
CKPNETFQIGCNRCRCNSEGTLYSCTRVGC
>BMPD-11
CQPGQEFRLDCNKCLCDKEGKDFSCTRMDC
>BMPD-12
CVPGSVYNQGCNVCRCTDEGRHATCTLMRC
>BMPD-13

CNPGEQFTRDCNDCTCSADGKSVFCTLRLC

Antheraea mylitta – Saturniidae - Lepidoptera
>AMPD-1
CEPGTHWKEGCNFCYCVEAGIPACTTLLC
>AMPD-2
CLPGTTWKSQCNYCLCLGDGYPACTFKEC
>AMPD-3
CAAHTIWRDECIVCRCTIYGEASCSMLAC

Samia cynthia ricini – Saturniidae - Lepidoptera
>SCPD-1
CLPRSKWESNCHSCECSESGAAKCVKQKDC
>SCPD-2
CKPNTTFNKGCNTCLCLENGLGLCTLQNC
>SCPD-3
CAPGSSWRSQCNDCVCNDQGISLCTLALC
>SCPD-4
CAPNTMWKNDCNTCRCTTAGKAMCTRIGC

Heliconius erato – Nymphalidae - Lepidoptera
>HEPD-1
CLAGSEWESLCHSCRCSEDGQPECARLGSC
>HEPD-2
CKPLTEFRRDCNICICFESGLGSSCTSMEC
>HEPD-3
CKVGTKWQSQCNNCSCSDDGMPMCTDMAC
>HEPD-4
CALGTSWKLDCNTCYCPPTGRAACTRIGC
>HEPD-5
CRPDEVFEVDCNMCRCSVDGMSFSCTRRAC
>HEPD-6
CQPGQEFTMDCNKCLCNNEGQDYSCTRINC
>HEPD-7
CVPGSVFKQGCNTCQCTEDGNHATCTIKRC
HEPD-8
CNPGEQFKRGCNDCACSADGKSVFCALRLC

Danaus plexippus – Nymphalidae - Lepidoptera
>DPPD-1
CNKCLCDNEGQNFSCTRIDC
>DPPD-2
CTPGSVFTQDCNTCRCTEDGGHATCTLKQC
>DPPD-3
CNPGEQFKRDCNDCTCSANGRGVFCTLRIC
>DPPD-4
CSVVAFILCLATFCRGGVIKCTPGSKDETC
>DPPD-5
CSPRTEWKSKCHRCICSDSGQALCFKIEGC
>DPPD-6
CKPESKFSRDCNSCLCTNNGNVICTLKAC
>DPPD-7
CAANRMFIKDCNTCWCNEDGTSFFCTRKVC

Manduca sexta – Sphingidae - Lepidoptera
>MSPD-1
CQPGSEWESNCHSCKCSEAGIAECLKQAAC
>MSPD-2
CKPNTNVAEGLPHLHLSGRRIRLCTLKNC
>MSPD-3
--QETSWMNECHHCWCTSKGYRACTLKGC

>MSPD-4
FHDPEPQHFLEEKCYICPLFYSGAAFCPLMEC
>MSPD-5
CEPRAEFKSECNVCKCSADGRSFSCTQNEC
MSPD-6
CQPNTIFYVACNACQCNNQGTDFACTLKIC

Epiphyas postvittana – Tortricidae - Lepidoptera
>EPPD-1
NSCTCLENGLGLCTLKNC
>EPPD-2
CAPGSEWKSGCNDCVCTPEGLRSCSSAGC
>EPPD-3
CALHSLWNKESNTCWCTSDGRAMCTKIGC

Hymenoptera

Apis melifera – Apidae - Hymenoptera
>AMPD-1
CVPGKSFFDGCNTCTCTDDGNFICTMTAC

Nasonia vitripennis – Pteromalidae - Hymenoptera
>NVPD-1
CFPGAVFQDDCNGCICGSDGKATCTNMDC
>NVPD-2
CVPGSELIHRCNQCFCTDSGTAMMCFKMGC
>NVPD-3
CQADKIFDYNCHQCICDAKGNYAMCSGKEC
>NVPD-4
CNPGMIFASDCNVCICSKNGKGVCTTFSC
>NVPD-5
CLPGSVFLQDCNACTCSNDGLSAACTDMAC
>NVPD-6
CEPSTVFKVYCNTCGCSSDGSSFSCTRMAC
>NVPD-7
CEPRTQFKEYCNTCGCADDGLSYICTRRMC
>NVPD-8
CKPHSNFKDYCNTCFCNNDGSEFACTRMSC
>NVPD-9
CEPRSHFKDYCNTCACSEDGTTYGCTMMMC
>NVPD-10
CTPGQVFFMSCNLCKCSSDGNYAACTFMQC
>NVPD-11
CPSKSFYNDCNMCVCGPDDASAACTMMMC
>NVPD-12
CPAGEFFHDKCNVCHCSANGFSAACTLMGC
>NVPD-13
CTPGSTFQMDCNSCTCSNDGKTAMCTGIAC
>NVPD-14
CTPGSNFHQDCNSCICLKDGQSAMCTGIAC
>NVPD-15
CVPKSKFNDYCNTCGCSDDGSSFICTRRLC
>NVPD-16
CKPRHLFKKDCNHCVCNAGGETAQCTVLDC
>NVPD-17
CVPGKSYFDGCNTCFCSEAHSVQCTRRLC
>NVPD-18
CPANQPFKWNCNYCTCGPEGKDASCTRMAC
>NVPD-19
CPPNESFMDKCNYCRCGPEGKDAACTKMNC
>NVPD-20

CTPGTYFKTECNTCVCAKDGSASICTQKQC
>NVPD-21
CTPNSFFHINCNRCQCSKHGIQYECEKNAC
>NVPD-22
CEPNQVFQNNCNTCACNKDSTAAACTLKEC

Nasonia Giraulti – Pteromalidae - Hymenoptera
>NGPD-1
CVPGKSYSDGCNTCFCSEAHSVQCTRRLC
>NGPPD-2
CPANQSFKWNCNYCTCGPEGKDASCTRMAC
>NGPD-3
CPPNESFIHKCNYCKCGPEGKDAACTKMNC
>NGPD-4
CKPKTRFKFYCNTCWCSEEGTTRICTKKYC
>NGPD-5
CKPKTRFKFYCNTCWCSEEGTTRICTKKYC

Pimpla hypochondriaca – Ichneumonidae - Hymenoptera
>PHPD-1
CEIGSNFKNYCNNCYCFDGVMDHALCTRESC
>PHPD-2
CTPGENFKYYCNDCQCLDGLRAHAMCTRMRC
>PHPD-3
CAPGASFKYYCNSCTCGAEGKVAEAQCTSQEC

Solenopsis invicta – Formicidae - Hymenoptera
>SIPD-1
CIPGSSYFDGCNNCFCQNDAHTVGCTTNWC

Hemiptera

Myzus persicae - Aphididae - Hemiptera
>MPPD-1
CDPGELVFVGFCNLCLCNSQGIPNQLCARSWC

Acyrthosiphon pisum – Aphididae - Hemiptera
>APPD-1
CDPGELVFVGFCNLCMCNSQGMPNQLCARSWC

Nilaparvata lugens – Delphacidae - Hemiptera
>NLPD-1
CLPGELVWVKCNLCLCNLEGQPNAVCAKMWC

Rhodnius prolixus – Reduviidae - Hemiptera
>RPPD-1
CKPGSVWKEDCNTCFCTETGHVGCTLMHC
>RPPD-2
CTPGTTWKEDCNTCFCSSTGQIGCTLMAC

Triatoma infestans – Reduviidae - Hemiptera
>TIPD-1
CEPNTRFKQECNWCTCSANGEYATCTLLYC
>TIPD-2
CGFGGTVPAGDGCNFCFCTPLGTIGTCTMRRC

Oncopeltus fasciatus – Lygaeinae - Hemiptera
>OFPD-1
CTPGSSWREKCKSCVCSKNGTPSCTRILC

Oncometopia nigricans – Cicadellidae - Hemiptera

>ONPD-1
CVPGTTWMDDCNTCHCNVNGLAACTRMLC
>ONPD-2
CTPGSSWTENCQTCTCSDQGAVTCTGDSC

Graphocephala atropunctata – Cicadellidae - Hemiptera
>GAPD-1
CVPGTTWKNDCNSCFCSETGVAACTLMGC
>GAPD-2
CTPGSTWTDNCQSCTCSEEGKASCTGAPC

Phthiraptera

Pediculus humanus – Pediculidae - Phthiraptera
>PGPD-1
CEPGQSFAKECNTCTCPDSGLKSLAGCTLKLC
>PHPD-2
CEPGSTFKMECNSCKCTDDGTDMVCTKKLC
>PHPD-3
CTPGETFKKLCNDCTCPPTGHKSAATCTLLTC
